# Uptake and Fate of Extracellular Membrane Vesicles: Nucleoplasmic Reticulum-Associated Late Endosomes as a New Gate to Intercellular Communication

**DOI:** 10.3390/cells9091931

**Published:** 2020-08-21

**Authors:** Denis Corbeil, Mark F. Santos, Jana Karbanová, Thomas Kurth, Germana Rappa, Aurelio Lorico

**Affiliations:** 1 Biotechnology Center (BIOTEC) and Center for Molecular and Cellular Bioengineering (CMCB), Technische Universität Dresden, Tatzberg 47-49, 01307 Dresden, Germany; jana.karbanova@tu-dresden.de (J.K.); 2 College of Osteopathic Medicine, Touro University Nevada, 874 American Pacific Drive, Henderson, NV 89014, USA; mark.santos@tun.touro.edu (M.F.S.); germana.rappa@tun.touro.edu (G.R.); 3 Center for Regenerative Therapies Dresden and CMCB, Technische Universität Dresden, Fetscherstraße 105, 01307 Dresden, Germany; thomas.kurth@tu-dresden.de (T.K.); 4 Mediterranean Institute of Oncology, Via Penninazzo, 11, 95029 Viagrande, Italy

**Keywords:** exosome, extracellular vesicle, intercellular communication, late endosome, oxysterol-binding-related protein, nucleoplasmic reticulum, Rab7, VAMP-associated protein A

## Abstract

Extracellular membrane vesicles (EVs) are emerging as new vehicles in intercellular communication, but how the biological information contained in EVs is shared between cells remains elusive. Several mechanisms have been described to explain their release from donor cells and the initial step of their uptake by recipient cells, which triggers a cellular response. Yet, the intracellular routes and subcellular fate of EV content upon internalization remain poorly characterized. This is particularly true for EV-associated proteins and nucleic acids that shuttle to the nucleus of host cells. In this review, we will describe and discuss the release of EVs from donor cells, their uptake by recipient cells, and the fate of their cargoes, focusing on a novel intracellular route wherein small GTPase Rab7^+^ late endosomes containing endocytosed EVs enter into nuclear envelope invaginations and deliver their cargo components to the nucleoplasm of recipient cells. A tripartite protein complex composed of (VAMP)-associated protein A (VAP-A), oxysterol-binding protein (OSBP)-related protein-3 (ORP3), and Rab7 is essential for the transfer of EV-derived components to the nuclear compartment by orchestrating the particular localization of late endosomes in the nucleoplasmic reticulum.

## 1. Introduction

Intercellular communication is a fundamental feature for the development and maintenance of multicellular organisms. Diverse molecular mechanisms for the exchange of biological information between cells have been documented. The secretion of soluble proteins and their interaction with membrane receptors located on the target cells or contact-dependent signaling are good examples. To better understand the mechanism that triggers molecular crosstalk between cells, we came to the fascinating and poorly explored world of extracellular membrane vesicles (EVs) [1,2,3,4]. We will review here the mechanisms underlying the release of different types of EVs from donor cells into the extracellular medium, their uptake by recipient cells, and the fate of their cargoes with a focus on a new intracellular pathway that led to their transfer into the nuclear compartment [5,6].

## 2. Extracellular Membrane Vesicles and Intercellular Communication at a Glance

EVs are nanobiological membrane structures usually referred to as exosomes or microvesicles depending on their biogenesis and size [7,8]. Exosomes (~40–100 nm in diameter) are produced by inward budding inside an endosome, leading to the formation of a late endosomal multivesicular body (MVB) that could fuse afterward with the plasma membrane and discharge its internal small vesicles in the extracellular milieu, whereas microvesicles, also named ectosomes or shed vesicles (~50–1000 nm), bud directly from the plasma membrane. To complete our non-exhaustive view of the EV catalog, in addition to apoptotic microvesicles (<1000 nm) and apoptotic bodies (1–5 μm) derived from dying cells [9], bulky EVs (1–10 μm), often termed large oncosomes [10], are formed upon cleavage of plasma membrane extensions of tumor cells harboring an amoeboid-like phenotype [11,12]. Nodal vesicular parcels involved in left–right asymmetry during the organism development can be viewed as atypical particles or EVs (~300–500 nm) containing lipophilic granules [13]. Lipoprotein particles can also contribute to exchange materials and promote signaling between cells [14]. Hereafter, we will focus on exosomes and microvesicles.

EVs carry a restricted set of membrane-protected proteins, lipids, and nucleic acids (e.g., messenger (m) RNA, micro (mi) RNA, and long non-coding (nc) RNA) that could act as pivotal mediators in the regulation of neighboring and distant recipient cells [15,16,17,18,19,20]. The number of bioactive molecules carried by a given type of EVs, especially small-sized ones such as exosomes, can be extremely little; therefore, an efficient mechanism must operate to trigger a cellular response. This is particularly true when the target cells are distant from the donor cells and the amount of EVs is limited [21,22]. The content of EVs depends on the cell type of origin and their physiological conditions. Thus, EVs represent a heterologous population in a given biofluid. Specific isolation of their subpopulations is now an emerging challenge in the field, particularly when the purpose is to use EVs (or their cargo) as potential clinical biofluid markers [23,24,25,26,27,28,29]. We could not exclude that distinct EVs interact with each other, resulting in their co-purification [30]. All these pitfalls should be considered. Diverse biological functions are ascribed to EVs in cell-to-cell communication, such as favoring proliferation versus differentiation of stem cells, inducing epithelial-mesenchymal transition, and modulating immune responses among others [31,32,33]. EV-mediated intercellular communication within an organ is also beginning to be recognized. For instance, neural cells exchange biochemical information upon the release of exosomes or microvesicles [34]. In addition to inter-neuronal communication, EVs have been suggested to play a role in crosstalk between neurons and glial cells, notably oligodendrocytes, and hence support the neuronal physiology [35,36]. In pathological conditions, EVs might participate in intercellular transfer of prions or the progression of various diseases including Alzheimer’s disease [37,38,39]. In cancer, the components carried by transformed cell-derived EVs play a role in the establishment of a pre-metastatic niche [40,41,42,43]. They can also contribute to horizontal propagation of oncogenes among subsets of cancer cells or surrounding healthy cells [44,45]. Lastly, the EV-mediated crosstalk between cancer and non-cancerous cells in the bone marrow microenvironment can modulate the biochemistry and function of stromal cells to stimulate the growth and spreading of cancer cells [46,47,48].

### 2.1. Release of Extracellular Membrane Vesicles

**Figure 1:**
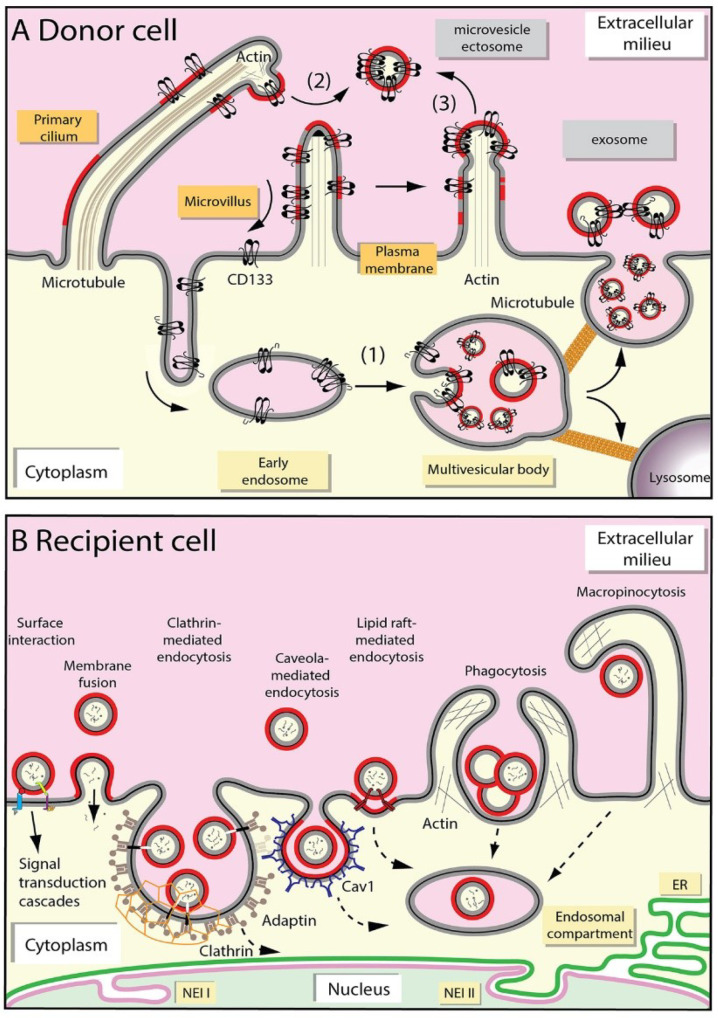
Mechanisms of the release of EVs by donor cell and their uptake by recipient cell. (**A**) The release of EVs from a healthy donor cell can occur by two major pathways that are not mutually exclusive. CD133^+^ EVs can be discharged into extracellular milieu upon fusion of multivesicular body (MVB) with plasma membrane. They will be referred to as exosomes. The internalization of CD133 and its transport to the early and late endosomal compartments can be mediated by its ubiquitination and interaction with syntenin-1. The biogenesis to small intraluminal vesicles within late endosome/MVB involves an inward invagination and budding of the limiting membrane of the endosome toward its own lumen (1). MVB could also fuse to lysosomes, leading to proteolytic degradation of CD133 and other proteins. The endosome movement requires a microtubule network. Alternatively, CD133^+^ EVs can bud from plasma membrane, notably protrusions such as primary cilium (2) or microvilli (3). They will be referred to as microvesicles or ectosomes. Cholesterol-dependent membrane microdomain (red membrane segment) can play a role in these budding processes. (**B**) The transfer of EV cargoes to the neighboring or distant recipient cell can occur by several mechanisms. The receptor-mediated binding of EVs to recipient cell could promote a signaling cascade, and hence elicit a pleiotropic response in recipient cells. Fusion of EVs with plasma membrane might occur, and consequently release randomly their content into cytoplasm of host cells. In addition, recipient cells can internalize EVs en route to intracellular molecular targets. Various mechanisms of endocytosis were reported. Clathrin-dependent mechanism required adaptins, which connect membrane cargo to clathrin forming a polyhedral lattice that surrounds the vesicle. Caveolin-1 (Cav1) plays a role in caveola-mediated uptake. Caveolae have a flash-shaped structure and are enriched in cholesterol and sphingolipids. Other lipid raft-mediated EV internalization processes independent of clathrin and caveolin are also proposed models of endocytosis. Phagocytosis and macropinocytosis are alternative mechanisms implicated in the internalization of EVs (see main text for details). After endocytosis, the EV content can be transferred to the cytoplasm of the recipient cells upon fusion of EVs with endosomal membrane and/or transported to nucleus (Figure 2 and Figure 3). The nucleoplasmic reticulum can be involved in the latter process. Both types (I and II) of nuclear envelope invaginations (NEI) are depicted. In type I NEI, only the inner nuclear membrane (INM) penetrates into nucleoplasm, whereas in type II both the INM and outer nuclear membrane (ONM). The endoplasmic reticulum (ER) is an extension of the ONM, and consequently, both membranes share certain constituents.

In line with a variety of EV subtypes, numerous modes of EV release were described [49]. Our labs gained insight into mechanisms underlying EV secretion by following the cellular trafficking of the stem cell marker CD133 (prominin-1), which is associated with plasma membrane protrusions (e.g., microvilli and cilia) and regulates their organization and function [50,51,52]. Although the observations with CD133 are not exclusive to this glycoprotein, they summarized the knowledge about the EV biogenesis. CD133^+^ EVs are released into various internal and external body fluids using different mechanisms that are dependent in part on the cellular type [53]. In non-epithelial cells, CD133^+^ EVs are discharged as exosomes via exocytosis of MVB (Figure 1A, point 1) [54]. The interaction of CD133 with syntenin-1 and its ubiquitination might be two essential steps involved in the internalization and sorting to MVB en route to exosomes [55,56]. Together with ALIX and syndecan, syntenin-1 regulates the biogenesis of exosomes [57]. The ubiquitinated CD133 interacts with tumor susceptibility gene 101 protein (TSG 101), a component of endosomal sorting complex required for transport (ESCRT) machinery involved in MVB formation [56]. We invite the readers to consult the following excellent reviews [3,58,59,60] for mechanistic details related to (i) the sorting of proteins to exosomes; (ii) the MVB biogenesis including ESCRT machinery; and (iii) their fusion with plasma membrane (Figure 1). In epithelial cells, CD133^+^ EVs bud as microvesicles/ectosomes from primary cilium and/or microvilli present at the apical plasma membrane (Figure 1A, points 2 and 3) [61,62]. Nader and colleagues described that actin polymerization is required for the release of microvesicles from ciliary tips by promoting membrane scission [63]. CD133 could itself play a role in this process [51,64]. ESCRT complex proteins might also participate in the release of microvesicles from plasma membrane [65]. The midbody that bridges nascent daughter cells at the end of cell division is also a source of microvesicles, and the midbody itself can be released depending on cellular status [61,66].

Lipid-based membrane microdomains (known as lipids rafts) and/or certain lipids (e.g., cholesterol, and ceramides), lipid-metabolizing enzymes, and lipid-interacting proteins may play a role in initiating budding processes either at the level of endosomes or plasma membrane (Figure 1A, red segment) [51,54,67,68]. The enrichment of lipid raft-associated cholesterol and sphingomyelin in EVs is consistent with such hypothesis [24,69]. Tetraspanin proteins such as CD9, CD81, and CD82 could be implicated in the sorting of various cargoes to EVs by forming a dynamic platform within the lipid bilayer membrane with other membrane and cytosolic proteins that would favor the formation of a specific dynamic network (also known as the tetraspanin web), and hence stimulate the budding process [70,71,72]. To conclude, it is important to note that the release of EVs by a given cell type is not restricted to a particular pathway; however, one mechanism can predominate under specific physiological or pathological conditions.

### 2.2. Uptake of Extracellular Membrane Vesicles

Once released in biofluids or the extracellular milieu between cells embedded in a tissue, circulating EVs can spread the selective information that they carry. All cells that are in direct contact with a given biofluid containing EVs are potential targets. Although poorly characterized, the diffusion of EVs within a tissue might be regulated by a source-sink mechanism, as suggested for morphogens [73,74]. Several barriers can impede the proper delivery of EVs such as their sequestration in the extracellular matrix, their degradation by proteolytic enzymes, and/or unspecific binding to non-targeted cells. The interaction of EVs with cell surface-associated extracellular matrix in a given organ/tissue may allow the specific targeting of EVs, a process mediated by EV-associated specific integrins, and their uptake by resident cells at the predicted metastatic destination [75,76]. The integrins (e.g., β1) carried by EVs can also promote anchorage-independent growth of tumor cells [77,78].

To achieve their vehicle-like function, EVs need to interact with recipient cells, which can be done in certain cases in a specific manner [79]. EVs could bind directly to the cell surface of recipient cells. In kidney, cellular protrusions such as cilia are the preferential sites for EV binding [80]. Exosomes can also “surf” on filopodia to enter cells at specific endocytic hot spots [81]. Molecular bases for the selective cellular targeting of EVs are poorly described [82,83]. The initial cellular binding of EVs can be mediated by adhesion proteins, and several classes of mediators are known to promote EV-cell interaction such as tetraspanins, lectins, heparan sulfate proteoglycans, and certain lipids [60,84,85,86]. A well-established example is the targeting of EVs (exosomes) to dendritic cells, which is mediated by tetraspanins (CD9, CD81), milk fat globule-E8/lactadherin, CD11a, and CD54/intercellular adhesion molecule 1 present on exosomes and α_v_/β_3_ integrin, CD11a, CD54 present on dendritic cells [87]. As reported for apoptotic bodies or certain viruses, phosphatidylserine present at the outer leaflet of EV membrane can play a role in their cellular entry [88,89,90]. We showed that monovalent antibody against CD9 can impede the entry of EVs into melanoma cells [91]. Further efforts are needed to dissect all mechanisms regulating the specificity of EV-recipient cell interactions, which is an important step to design new strategies based on EVs as a bio-vehicle for therapeutic drug delivery [92,93,94].

Several mechanisms that are not mutually exclusive were proposed to explain the transfer of bioactive molecules between EVs and recipient cells (Figure 1B) [95,96]. The machinery responsible for the transmission of information and/or cellular entry of EVs might be determined by composition of EVs and the plasma membrane of recipient cells, as well as the size of EVs or their aggregation [97]. Receptor-mediated binding of EVs, or of EV-derived soluble ligands, to recipient cells could promote a downstream signaling cascade and elicit a pleiotropic response [95,98,99,100,101]. Fusion of EVs with the plasma membrane and hence the direct release of their content into cytoplasm might occur [102]. An acidic milieu as observed in cancerous tissue microenvironment might favor EV-cell fusion [103]. The acidity-mediated direct fusion of EVs with tumor cells may be similar to the fusion of endocytosed EVs with limiting late endosomal membranes observed in the low pH endocytic compartment and/or the entry of certain enveloped viruses [104,105,106]. Under these extreme conditions, alterations in membrane fluidity and/or unmasking of fusogenic proteins may account for the fusion ability [103]. Tetraspanin-rich microdomains and/or lipid rafts present within the EV membrane and/or the plasma membrane of recipient cells may facilitate their fusion [107,108].

In addition to these two processes that trigger a cellular response, recipient cells can internalize EVs en route to intracellular targets [109]. The literature about EV uptake by endocytic mechanisms is growing, but sometimes conflicting [110]. Various mechanisms of endocytosis were described, including clathrin-dependent, caveolae, and/or cholesterol-rich lipid rafts mechanisms (Figure 1B) [95,111,112,113,114,115,116,117,118]. Clathrin-dependent endocytosis involves the engulfment of receptors associated with their ligands in clathrin-coated pits on the plasma membrane that invaginate and form clathrin-coated vesicles. Treatments affecting the formation and/or dynamics of clathrin-coated pits can impede the EV entry [114,119]. Caveolar endocytosis is based on the properties of caveolin proteins to oligomerize, which leads to the formation of caveolin-rich microdomains within plasma membrane ending to the endocytic pathway, notably late endosomes [120]. In early days, this specialized caveolin-rich endosomal compartment was named “caveosome”, which turned out to be “artifactual” endosomes somehow produced by caveolin overexpression [120,121,122,123]. Caveolins are cholesterol-binding integral membrane proteins with unusual membrane topology where their uniquely long hydrophobic segment does not span the membrane bilayer. Other players involved in caveola architecture are members of Cavin family, i.e., peripheral membrane proteins that coat the caveolar surface [124,125]. In addition to caveolins and cavins, caveolae contain dynamin, a GTPase also involved in the biogenesis of clathrin-coated vesicles which plays a role in pinching off the caveolar vesicle [123,126]. Similar to lipid raft domains, caveolae are rich in cholesterol and sphingolipids, and membrane cholesterol is essential for their formation [127]. Cholesterol-rich lipid rafts can also promote endocytosis independent of the presence of caveolae or clathrin coats [128]. Lipid raft-associated flotillins and annexins can play a role in such endocytic pathways [129,130]. Ruffling of the plasma membrane of recipient cells can also lead to the internalization of large volumes of extracellular fluid containing EVs, a phenomenon referred to as macropinocytosis [131,132]. Cell type-specific phagocytosis is an alternative way for EV uptake. In these processes, an active remodeling of the actin cytoskeleton and the participation of phosphatidylinositol 3-kinase are essential (Figure 1B) [114,133].

### 2.3. Fate of Extracellular Membrane Vesicles upon Internalization

Despite current knowledge on the biogenesis and uptake of EVs and their relevance in various medical areas as biomarkers, therapeutic targets, or biological vehicles for drug delivery, information on the fate of endocytosed EV content remains limited [21,22]. The heterogeneity of EVs and the mechanism of cellular internalization can determine the fate of EV content. Various pathways can operate simultaneously, leading to various effects. Upon endocytosis, internalized EVs traffic to the early endocytic pathway. Little is known about the role or fate there. If a fusion occurs with the early endosomal membrane, their soluble cargo would reach the cytoplasm, while EV-associated membrane proteins could potentially travel to the trans-Golgi network, Golgi complex, and endoplasmic reticulum (ER) via retrograde transport. They can also reach the plasma membrane through recycling endosomes. To our knowledge, however, such EV-early endosome fusion was never reported. Movement of endocytosed EVs from early endosomes to the late endosomes/MVB, and again their discharge into the extracellular milieu upon MVB-plasma membrane fusion, is a potential avenue [134]. Although this recycling mechanism appears futile in regard to the fate of cargo carried by EVs, it could nonetheless allow transfer of EV-associated components through a cell en route to the proper recipient. The transcytosis through a cellular barrier such as an epithelium and/or a blood vessel might allow EVs and/or their specific content to reach distinct cells. Alternatively, EV-derived components can be sent to lysosomes for degradation [135]. Such endosomal-lysosomal degradative pathway leading to EV clearance would need further thought to understand how it could elicit a cellular response. Nonetheless, the EV loading of such degradation pathway might indirectly influence the fate of other intracellular routes (e.g., autophagy), and hence the final destination of their components [136,137].

As communication vehicles, the endocytosed EVs can fuse with late endosomes and release their soluble content directly into the cytoplasm of the host cells [138,139], while their membranous components would mix with those of endosomal membranes. The acidic pH in the late endosome microenvironment would favor such fusion, as discussed above. As recently underlined by Mathieu and colleagues, endocytosed EVs and/or degradation products therefrom can potentially escape the endocytic pathway upon rupture of endosomal/lysosomal membrane [97]. Further investigations are required to decipher the spatiotemporal breakdown of late endosomes, and how this potential mechanism can be coordinated with EV function as a messenger of information. In any case, the subcellular localization of late endosomes (e.g., peripheral areas versus perinuclear) can indirectly regulate the interaction of EV cargo molecules with cellular targets [140]. In this regard, we recently described a new intracellular pathway that led to the transfer of the EV content, notably transmembrane proteins and nucleic acids, to the nuclear compartment of host cells. Nuclear transfer of EV cargo was monitored using engineered EVs expressing CD9-green fluorescent protein (GFP) fusion protein [5]. In a separate study, Read and colleagues reported nuclear transportation of exogenous epidermal growth factor receptor (EGFR) and androgen receptor via EVs [6]. Hereafter, we will present this novel intracellular path, the key organelles involved, i.e., nucleoplasmic reticulum and late endosomes, and the molecular players involved. The perspectives that this alternative intracellular route could bring to the medical field will be highlighted.

## 3. Nucleoplasmic Reticulum-Associated Late Endosomes: A New Gate to Intercellular Communication

### 3.1. Nuclear Envelope Invaginations

To grasp the novel intracellular pathway involved in the nuclear transfer of endocytosed EV-related cargoes, we need to introduce the nucleoplasmic reticulum, which is a complex branched network of tubular membrane-bound invaginations of the nuclear envelope that allows deep nuclear structures to be in the proximity of cytoplasmic components, thus increasing the interface between cytoplasmic and nucleoplasmic compartments. Although not all aspects of its biological function are fully understood, nucleoplasmic reticulum plays a role in calcium signaling in the nuclear compartment [141,142], and consequently in all features linked to it such as gene expression [143]. Two phospholipid bilayers, the inner nuclear membrane (INM) and outer nuclear membrane (ONM), form the nuclear envelope [144]. The narrow space between them is called perinuclear space. ONM is continuous with ER membrane and may share certain constituents (Figure 1B). At the nucleoplasmic side, a lamin-rich proteinaceous meshwork underlies the INM. Nuclear envelope invaginations (NEI) of type I are formed solely by INM penetrating in the nucleoplasm, while type II are formed by both INM and ONM (Figure 1B) [145]. The latter can form channels throughout the nucleus and contain nuclear pores [146,147]. Often, they are in close contact with nucleoli. Type II NEI are enfolded around intermediate filaments, microtubules, and possibly actin cables that are connected to the ONM, and hence link the nuclear compartment to the cellular microenvironment [145,146,148]. The presence of cytoskeleton elements within NEI might be regulated by the cellular status, just like NEI themselves [149]. In certain type II NEI, such as those detected in stressed or polyploid cells, ribosomes and mRNA translation machinery were observed therein [150], which is consistent with the continuity between ONM and ER.

The biogenesis and dynamics of NEI are observed in interphase nuclei [151]. Recently, Vaux and colleagues demonstrated that the formation of the nucleoplasmic reticulum requires the de novo assembly of new components, i.e., nascent membrane phospholipids and lamins, rather than a simple reorganization of the pre-existing nuclear envelope [152]. The protein players involved in the membrane shaping of tubular invaginations during their biogenesis, and possibly their remodeling, remain to be identified. Enzymes involved in membrane lipid metabolism and components of nuclear pores deserve particular attention in this respect [153,154,155]. Other potential mechanisms explaining the formation of tubular invaginations of the nuclear envelope cannot be ignored [156]. Thus, they can be created by dynamic chromatin movements resulting from the interaction of chromatin-INM-associated proteins by pulling the nuclear envelope into the nucleoplasm [157]. Alternatively, the pressure created by cytoplasmic-associated cytoskeleton elements on nuclear membrane can create type II, but not type I NEI (see below). The abundance of NEI seems linked to the status of cellular differentiation; thus, Johnson and colleagues suggested that NEI indicate the degree of cellular de-differentiation [149]. In cancers, the number of NEI is significantly elevated and can be used for their classification [158,159]. Given that NEI are widely distributed among cells under normal and pathological conditions, they deserve further attention.

### 3.2. Nucleoplasmic Reticulum-Associated Late Endosomes 

**Figure 2:**
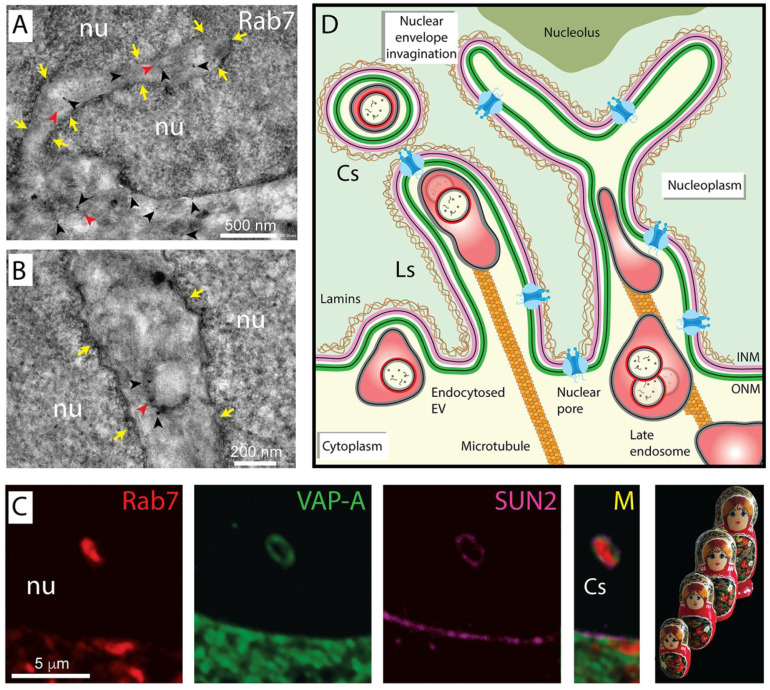
Entry of late endosomes into type II nuclear envelope invagination. (**A**,**B**) The presence of membrane-bound organelles (red arrowheads) in the nucleoplasmic reticulum (arrows) and cytoplasm of melanoma FEMX-I cells are observed by Rab7 immunogold labeling (black arrowheads) as described [160]. [Anti-Rab7 rabbit monoclonal antibody D95F2, goat-anti-rabbit Nanogold (1 nm), and silver enhancement were applied]. (**C**) HeLa cells expressing Rab7-red fluorescent protein were double immunolabeled for VAP-A (outer nuclear membrane (ONM) marker, green) and SUN2 (inner nuclear membrane (INM) marker, magenta) [160]. A cross-section of nuclear envelope invagination (NEI) containing Rab7^+^ late endosomes is displayed. Note the presence of various membranous structures, e.g., the double-layered membrane of the nucleus and the membrane of Rab7^+^ late endosomes—the latter might contain intact endocytosed EVs. Their arrangement one inside another (M, merge) has some similarity to Matryoshka dolls. (**D**) Representation of type II NEI that contain late endosomes. Some late endosomes are depicted with endocytosed EVs, which themselves contain cargo molecules. An intact microtubule network is essential for the translocation of late endosomes in a NEI that often lies close to nucleolus. A lamin-rich proteinaceous meshwork underlies the INM. Cross-sections (Cs) and longitudinal (Ls) sections of NEI. Nu, nucleoplasm. [Fluorescence images were originally published in the Journal of Biological Chemistry: Santos MF, Rappa G, Karbanová J, et al. VAMP-associated protein-A and oxysterol-binding protein-related protein 3 promote the entry of late endosomes into the nucleoplasmic reticulum. 2018; 293:13834–13848 [160]. ^©^The American Society for Biochemistry and Molecular Biology].

In search for the mechanism regulating the transfer of biomaterials from EVs to their molecular targets in recipient cells, notably in the nuclear compartment, we observed that a fraction of EV-associated proteins, upon EV endocytosis, ends in type II NEI, suggesting that the endocytic system can be involved in nuclear transfer [5,160]. The literature has documented the presence of membrane-bound vesicles or organelles in NEI in addition to cytoskeleton elements [145,146]. For instance, Lui and colleagues described the presence of autophagosome-like multi-lamellar bodies in NEI [141,161]. From our side, we have recently demonstrated by immunogold electron microscopy and confocal microscopy [5,160] that a subpopulation of late endosomes labeled with small GTPase Rab7 was associated with type II NEI (Figure 2A–C). As observed on longitudinal cross-sections of NEI, the frequently pseudo-elongated morphology of late endosomes therein resembles a sword in its scabbard [5], which led to propose the name of “*spathasome*” (from “*spathi/spatha*” (Greek/Latin) for sword) for this dual membrane-bound structure. The configuration of the double-membrane invaginations can be observed by immunofluorescence using the ER-associated vesicle associated membrane protein (VAMP)-associated protein A (VAP-A; see below), which also labeled ONM (Figure 2C, green), and INM-associated SUN domain-containing protein 2 (SUN2, Figure 2C magenta). Of note, the appearance of these nuclear membranes along the Rab7^+^ late endosomal membrane, one inside the other, has some similarity to stacking dolls (known as Matryoshka dolls; Figure 2C). Such resemblance becomes striking considering that the endocytosed EVs are present in late endosomes (Figure 2D) [5,160]. Since solely a minute fraction of EV-loaded late endosomes ended up in NEI in a steady-state condition, it remains to be determined whether they are derived from StAR-related lipid transfer domain containing 3 (STARD3)^+^ “early” or OSBP-related protein 1L (ORP1L)^+^ “late” late endosomes, as defined by the fluid-phase cargo transport [162]. STARD3 (also known as metastatic lymph node 64 (MLN64)) and ORP1L are two cholesterol-binding proteins. The former is a molecular tether transporting membrane cholesterol within ER–endosome contact and is involved in the egress of cholesterol from late endosomes/lysosomes to mitochondria [163,164,165], while the latter is part of a protein complex that includes Rab7 and regulates the minus-end transport of late endosomes [166]. Notably, neither STARD3 nor ORP1L proteins were present in NEI of melanoma cells, suggesting that late endosomes located there might represent a third subpopulation [5,160]. Additional markers are needed for their complete characterization. Given that the movement of late endosomes in nucleoplasmic reticulum is a dynamic process as observed by time-lapse video, we cannot exclude that a larger proportion of endosomes are moving there [5,160]. All the factors that govern this transfer are waiting to be discovered, as well as the limiting step it involves (see below).

In contrast to late endosomes, early Rab5^+^ endosomes were barely detectable in the NEI, as was the Golgi apparatus [5]. Mitochondria remained in most cases at the entrance of the NEI [5,145,146,167]. Under particular situations, mitochondria might shuttle in NEI and influence Ca^2+^ signaling [141,168]. It will be important to further dissect the types of membrane-bound structures that navigate in NEI, particularly in those making tunnels throughout the nucleus. Similarly, it would be of general interest to decipher whether subtypes of type II NEI with specific function(s) exist. The presence of inositol 1,4,5-trisphosphate (IP_3_) receptors in certain NEI suggests it [141,161].

### 3.3. Transport of EV-Loaded Late Endosomes in Nucleoplasmic Reticulum

**Figure 3:**
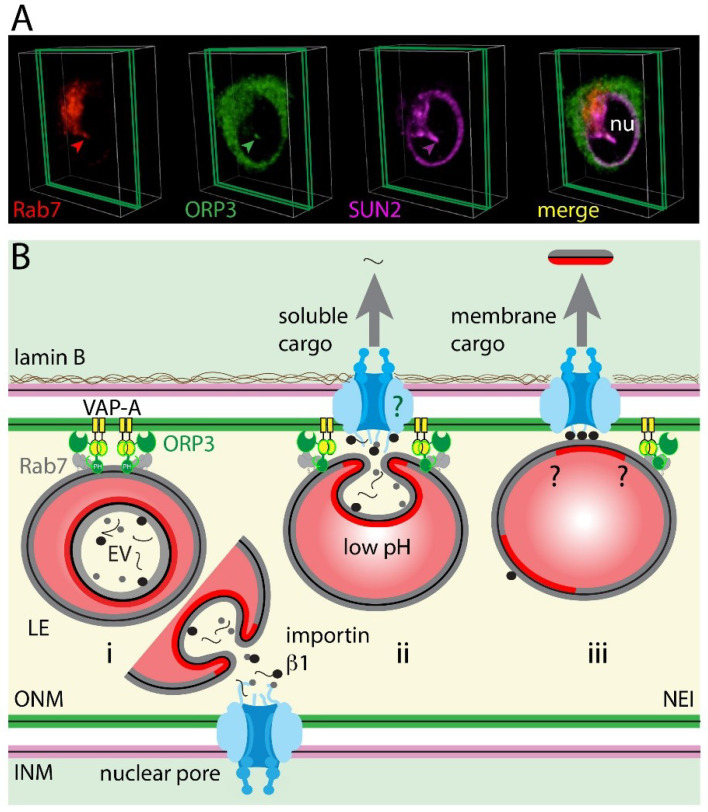
VOR complex is involved in the translocation of late endosomes into the nucleoplasmic reticulum and nuclear transfer of EV-derived components. (**A**) ORP3, a member of the VOR complex, is associated with nuclear envelope invagination (NEI). Melanoma FEMX-I cells expressing Rab7-red fluorescent protein were double immunolabeled for ORP3 and SUN2 as described [160]. 3D reconstruction of three optical sections (green slice) of a given cell is shown. The protein of interest within NEI is indicated (arrowhead). Nu, nucleoplasm. (**B**) Schema summarizing the players of VOR complex and their interactions within NEI. In this model, a late endosome containing endocytosed EVs (EV) is transported to NEI and tethered to outer nuclear membrane (ONM) (i). The latter process is mediated by the type II transmembrane, endoplasmic reticulum (ER)/ONM-associated VAP-A protein (yellow), cholesterol-sensor ORP3 protein (green) that binds to VAP-A via its FFAT motif, and late endosome-associated Rab7 (gray). The pleckstrin homology (PH) domain of ORP3 might mediate its binding to late endosomal membrane. The inhibition of transfer of EV-derived components (soluble and membranous) into the nucleoplasm of host cells after importazole treatment suggests that importin α1/β1 (may be those associated with EV; black spot) and nuclear pores play a role in these processes (ii). The potential interaction of nuclear pore components with VOR complex should be addressed in a near future (green question mark). Other urgent questions remain open, notably the mechanism(s) allowing the extraction of EV-derived membrane proteins (red) from endosomal membrane and their transfer into nucleoplasm through the nuclear pores, which are size restricted (iii, black question mark). The low pH milieu in late endosome might favor the fusion of endocytosed EVs with its membrane (i–iii). [Fluorescence images were originally published in the Journal of Biological Chemistry: Santos MF, Rappa G, Karbanová J, et al. VAMP-associated protein-A and oxysterol-binding protein-related protein 3 promote the entry of late endosomes into the nucleoplasmic reticulum. 2018; 293:13834–13848 [160]. ^©^The American Society for Biochemistry and Molecular Biology].

The mechanism regulating the translocation of late endosomes in type II NEI is an interesting topic that requires further exploration. The movement of late endosomes within the cytoplasmic compartment and their interactions with the ER membrane might be instructive. For instance, the integral ER-anchored protein protrudin contacts late endosomes via Rab7 binding and mediates their plus end-directed translocation along microtubules toward the cell periphery by the intermediate of the motor protein kinesin-1 and motor adaptor protein called FYCO1 (FYVE and coiled-coil domain containing 1) located on late endosomes [169,170]. The repeated contacts between late endosomes and ER membrane promote their translocation in a microtubule-dependent manner. Does a similar interplay mechanism occur in NEI? The presence of tubulin in type II NEI and the negative impact of nocodazole treatment on late endosome translocation to NEI support this hypothesis (Figure 2D) [160]. It might be more than a coincidence that protrudin binds via its FFAT motif (FFAT being an acronym for two phenylalanines (FF) in an Acidic Tract) to type II membrane protein VAP-A, and the knockdown of the latter impeded the subcellular localization of Rab7^+^ late endosomes in NEI of melanoma cells [160]. Although protrudin is not detected in all cell types including melanoma cells [160], the interaction of Rab7, Rab7 effector FYCO1 protein, and motor protein kinesin-1 could nevertheless mediate the nuclear translocation of late endosomes towards microtubules, as reported for the transport of autophagic vesicles [171]. As described for the positioning of late endosomes, an alternative mechanism involving the interactions of Rab7, ORP1L, Rab-interacting lysosomal protein (RILP), and dynactin subunit p150^Glued^, which binds to dynein heavy chain, could also explain the movement of late endosomes into NEI [140,172]. Although the cholesterol-sensor protein ORP1L, which interacts with VAP-A, is absent from NEI as mentioned above [160], other OSBP-related proteins of this large family could be involved [173]. Indeed, the OSBP-related protein-3 (ORP3), in contrast to STARD3 and ORP1L, was found in NEI and co-localized with Rab7^+^ late endosomes (Figure 3A) [160]. ORP3 knockdown, just like VAP-A, abolished the presence of Rab7^+^ late endosomes in NEI, indicating that this cholesterol-sensor protein could play a certain role in the late endosomes transport process and/or their tether to nuclear membrane within the NEI [160]. The next step will be to determine whether RILP and p150^Glued^ protein are associated with NEI and participate to the selective microtubule minus-end dynein-dynactin-dependent movement of late endosomes. A competition between FYCO1-kinesin and RILP-dynein-dynactin complexes for Rab7 binding and recruitment to late endosomes is conceivable.

The observation that, upon exposure to EVs, the proportion of cells harboring nucleoplasmic reticulum-associated late endosomes increased, and the proportion of cells without NEI decreased [5], suggests that the EV loading of the endosomal compartment regulates NEI biogenesis and consequently represents a new “pushing in” mechanism of their formation [5]. Thus, the translocation of late endosomes into NEI and the biogenesis of nuclear membrane folds can be two linked events involving microtubules. In addition to the presence of microtubules in NEI, microtubules running parallel to the nuclear envelope and contacting the cytoplasmic filaments of a nuclear pore complex have been observed by electron microscopy [174]. Nucleoporin proteins are indeed present in NEI containing EV-loaded Rab7^+^ late endosomes [5]. Nuclear pore proteins such as nucleoporin 358 (Nup358/RANBP2) and/or nuclear proteins such as SUN1/2 together with member(s) of the ONM-associated Nesprin protein family could anchor microtubules and somehow regulate NEI dynamics [175,176]. It is documented that SUN1/2 and Nesprin proteins play an important role in the nuclear envelope organization and nuclear positioning relative to the cell body [177,178,179,180]. By interacting with motor proteins (e.g., kinesin proteins such as KIF5B and KIF5C), they could anchor the microtubules within NEI, leading to a highway for the late endosome translocation [181]. These exciting issues await further investigation.

## 4. The VOR Complex: Interaction of VAP-A, ORP3, and Rab7

### 4.1. Inter-Organelle Contacts

The regulated interaction between membrane-bound organelles is an exciting and emerging field of cell biology [182,183]. The organelle crosstalk in eukaryotic cells plays an underestimated role in cell signaling and/or membrane dynamics. Exchange of biocomponents such as ions and lipids between connected organelles were described [184,185]. As a general controller of inter-compartmental activities, the ER, the largest organelle that forms a tubular network, appears to be determinant in numerous processes [186,187]. Contact sites between ER and Golgi apparatus, mitochondria, plasma membrane, peroxisomes, lysosomes, lipid droplets, and endosomes were documented [188,189,190]. Usually, the ER makes multiple contacts with a given organelle, bridging it in close proximity (i.e., less than 30 nm distance). As demonstrated by electron tomography, the distance between ER and late endosomes at the contact site is approximately 8 nm [191]. This short distance between connected organelles facilitates the non-vesicular membrane lipid transport, which requires specific proteins that can extract them from donor membrane, shield in a hydrophobic pocket, and transfer to opposite acceptor membrane. For mechanistic details about lipid transfer between opposite membranes and the involved lipid-transfer proteins, see the following reviews [173,187,192]. The repeated contact between organelles can also regulate the movement of one of them as discussed above, where late endosomes are translocated toward the cell periphery using ER and microtubules as support.

A certain number of molecular players involved in the contact zones between ER and various organelles were identified [186,193]. For instance, integral ER membrane-localized VAP-A and VAP-B (also known as ALS8) have been implicated in tethering and maintenance of structural and functional properties of the Golgi complex by regulating the proper levels of key lipids (e.g., phosphatidylinositol-4-phosphate, sphingomyelin, and diacylglycerol) in the Golgi membrane [194]. The knockdown of VAPs exerts various lipid-mediated effects on Golgi-mediated transport events. These actions are regulated by the interaction of VAPs with lipid-transfer/binding proteins, including PYK2 N-terminal domain-interacting receptor 2 (Nir2), OSBP, and ceramide-transfer protein (CERT). Their FFAT motif mediates the VAP interaction [195,196,197], while pleckstrin homology (PH) domain of VAP-interacting proteins promotes their binding to Golgi membrane [198,199]. FFAT-binding site in VAPs is associated with the major sperm protein (MSP) homology domain located at the cytoplasmic N-terminal part [200]. In addition to OSBP, the interaction of full-length ORP9 with VAPs also occurs and regulates trans-Golgi/trans-Golgi network structure and function by modifying its membrane environment by means of cholesterol delivery [201]. The contact between VAP-B and outer mitochondrial membrane protein, such as protein tyrosine phosphatase-interacting protein 51 (PTP1P51), regulates the ER-mitochondria association [202,203]. Direct contact between ER and plasma membrane was also described and implicated in various cellular processes such as regulation of intracellular calcium, lipid traffic, and signaling [204,205]. Among the scaffolding proteins involved, VAP-A and ORP3 were described [206]. Interestingly, ORP3 forms a physical complex with the small GTPase R-Ras [207]. The plasma membrane-targeting PH domain as well as VAP-A-interacting FFAT motif found in ORP3 were essential for the activation of R-Ras and its downstream signaling pathway, indicating that ER-plasma membrane contact creates a R-Ras activation platform [208]. Moreover, ORP3 interacts with IQSec1 (IQ motif and Sec7 domain-containing protein 1) protein, and the ORP3/VAP-A/IQSec1 complex plays a role in focal adhesion turnover [209]. Like other OSBP homologs, ORP3 was shown to bind to membrane cholesterol, as demonstrated using live cell photo-crosslinking with [^3^H] photo-cholesterol [210]. The endosomes are other organelles physically involved in the contact with ER. Indeed, ER participates actively in endosome maturation, transport, and fission [211,212,213]. The literature has documented various molecules implicated in the ER–endosome tethering, including paired proteins: VAP-A and STARD3 or STARDNL (STARD3 N-terminal like); VAP-A and ORP1L; protrudin/VAP-A and Rab7; and protein tyrosine phosphatase 1B (PTP1B) and endocytosed phosphorylated EGFR [193,214,215]. In the latter case, such inter-organelle contact (i.e., ER–endosomes) can regulate spatially and temporally EGFR signaling [216]. The interaction between VAP-A and endosomal proteins STARD3 and STARDNL could regulate endosome positioning and dynamics. In contrast to STARD3, STARDNL does not contain a START protein domain that allows cholesterol binding and can potentially transfer cholesterol between connected and adjacent membranes [217,218].

### 4.2. Late Endosome–Nuclear Membrane Contact: Role of VAP-A–ORP3–Rab7 Interactions

Applying a triple fluorescence analysis, fluorescence resonance energy transfer, and para-magnetic immune-isolation techniques, we demonstrated that VAP-A, ORP3, and Rab7 co-localize in discrete areas within NEI and interact with each other [160]. This tripartite complex, named VOR complex (an acronym for VAP-A, ORP3 and Rab7), is essential for the localization and docking of late endosomes in NEI (Figure 3B) [160]. Moreover, ORP3 needs VAP-A to be present in NEI, suggesting that VAP-A is orchestrating these interactions [160]. Surprisingly, the VAP-A homolog, VAP-B, is not involved in these events although it is also localized in nucleoplasmic reticulum [160]. Thus, VAP-A silencing cannot be rescued by VAP-B [160]. These findings highlight the most hidden part of molecular mechanisms controlling these sequential interactions (VAP-A/ORP3/Rab7) and their regulation. The hyperphosphorylation of ORP3 might promote the interaction with VAP-A via the FFAT motif as described for its role in ER-plasma membrane contact zone, while its PH domain could promote its interaction with late endosomal lipids [208,209,219]. In contrast to ORP1L, ORP3 does not contain an ankyrin repeat region, which could mediate Rab7 interaction [220]. It remains to be evaluated whether the R-Ras binding site in ORP3 can be involved [208]. Although the presence of Rab7^+^ late endosomes in NEI is fully abrogated after ORP3 silencing in melanoma cells [160], it will be of interest to evaluate whether other ORPs are engaged in the nucleus—late endosome contact in particular types of cells and/or under peculiar physiological and pathological conditions. A potential candidate is the ER-associated transmembrane protein ORP5, which has been suggested to interact with the late endosome cholesterol transporter Niemann-Pick C1 protein [221] (reviewed in [215]). The co-existence of multiple bridging complexes driven by distinct protein pairs cannot be excluded [204], and it remains to be identified whether other proteins participate in the tether of late endosomes with ONM within nuclear folds.

### 4.3. Implication of Nuclear Pores in EV-Derived Cargo Nuclear Shuttling

How can EV-associated components within late endosomes reach the nucleoplasm? After the docking of late endosomes to ONM, the fusion of EVs with the endosomal membrane might expose EV content in the proximity of nuclear pores, allowing their nuclear transfer. The presence of the nuclear transport receptor karyopherin (importin α and β1 subunits) in EVs [5,222,223,224,225] suggests that it may somehow participate in the nuclear translocation of soluble and perhaps membranous EV components (Figure 3B). The nuclear localization signal (NLS) present on a protein cargo is recognized by importin α1 subunit, which in turn interacts with β1. The resulting protein complex passes through the nuclear pore by binding key nucleoporins [226]. Once translocated into nucleoplasm, small regulatory nuclear GTPase Ran-GTP binds to importin β1 and dissociates the imported protein complex. Consistent with the role of nuclear pores in these processes, nucleoporins were detected in NEI containing late endosomes [5], and the incubation of cells with cell-permeable 2,4-diaminoquinazoline (importazole), a small molecule that inhibits the function of importin β1 by altering its interaction with Ran-GTP [227], impaired the nuclear translocation of EV-derived protein cargo [5]. The intriguing interaction of another ER-located OSBP-related protein, i.e., ORP8, with nucleoporin Nup62 might be relevant in this context [228]. A direct interaction between ORP3, together with VAP-A/Rab7, and nuclear pore constituents should be investigated (Figure 3B, question mark). Further, it remains to be determined how a protein complex notably those containing EV-derived membrane glycoproteins (e.g., full-length CD9 and CD133) are extracted from EV-endosomal membrane after the fusion of the former with the endocytic organelle [5]. Are tetraspanin-rich microdomains or lipid rafts present in the membrane bilayer of EVs involved in forming a phase separation with surrounding, more fluid membrane lipids (Figure 3B, double question marks)? Thus, tetraspanin/lipid raft-associated proteins might create a membranous platform, and NLS-containing transmembrane cargo/importin proteins could be extracted and drag the entire complex through the nuclear pore. Considering that nuclear pores are size restricted [229], we cannot expect that an entire endocytosed EV (>40 nm in diameter) reaches the nucleoplasm, although it was recently suggested that very small (nano)vesicles (<30 nm) other than high-density lipoprotein particles may exist [230]. The high degree of membrane curvature in NEI, particularly at their tip may alter the properties of nuclear pores and hence allow larger complexes to pass through. It remains to be investigated whether other pathways independent of nuclear pores are implicated in the nuclear translocation of EV-derived cargoes [231,232].

## 5. Biological Functions

The localization of late endosomes in the nucleoplasmic reticulum could have a significant impact on the mechanism of intercellular communication mediated by EVs. This novel endosomal pathway adds an alternative route to the soluble and membranous components derived from EVs (e.g., nucleic acids and proteins) to reach and interact with their molecular targets, including those associated with nucleoli [5,6]. This biological issue is particularly important given the limited amount of bioactive molecules carried by EVs. In addition to EV cargo, this pathway might play a role in the nuclear translocation of plasma membrane receptors upon ligand interaction. Similar to integral membrane proteins CD9 and CD133 [5,52,233,234,235], numerous plasma membrane receptors including EGFR, platelet-derived growth factor (PDGF), insulin growth factor receptor 1 (IGF-1R), and several G-protein coupled receptors that are potential drug targets were surprisingly found in the nuclear compartment [236,237,238,239]. Therein, they could be involved in transcriptional regulation and cellular proliferation, and confer chemo- and radio-resistance among other biochemical processes. In this context, it might be more than a coincidence that the nuclear translocation of the cell surface IGF-1R is dependent on the dynactin subunit p150^Glued^ and importin β1 as discussed above for the late endosome movement and nuclear transfer of EV cargo, respectively [238]. To date, the retrograde transport through Golgi and ER was considered the main intracellular route for full-length membrane-anchored proteins to the nucleus [240,241] (Table 1). Our observation with nucleoplasmic reticulum-associated late endosomes is in line with a recent pathway described by Chaumet and colleagues where the bacterial protein Pseudomonas exotoxin A upon binding to the cell surface LDL receptor related protein 1 (LRP1) can reach the nuclear compartment through docking and membrane fusion of early endosomes with the nuclear envelope [232] (reviewed in [242]). In the latter case, SUN1 and SUN2 proteins and Sec61 translocon complex were involved in this novel pathway (Table 1). Sec61 complex mediated the egress of proteins from INM to the nucleoplasm [232,242]. Although the mechanisms regulating these novel intracellular trafficking routes appear distinct, they nonetheless address the question of how cell surface and extracellular proteins reach the nucleoplasm. Potential mechanisms underlying the nuclear transports of membrane proteins present at the plasma membrane and/or EVs are summarized in Table 1.

In addition to the nuclear transfer of EV cargo, nucleoplasmic reticulum-associated late endosomes may create a privileged intracytoplasmic compartment that would favor protein–protein, protein–nucleic acid, and nucleic acid–nucleic acid interactions. The cytoplasmic release of soluble EV constituents upon a tardive fusion of intact endocytosed EVs with late endosomal membrane might facilitate their binding, given a local high concentration, to the molecular targets exported out of the nucleus such as RNA transcripts, and hence exert a function independent of their nuclear import [150]. This issue requires further attention.

## 6. Perspectives and Future Directions

Interference with the new intracellular route used by endocytosed EVs can find applications in the cancer field, for instance by preventing the delivery of cancer cell-derived EV cargo to host cell nucleoplasm. The identification of the VOR complex and its inhibition may be clinically relevant. Thus, disruption of the intercellular communication between cancer cells and stromal components within the metastatic niche may be a novel approach to intercept the mechanisms underlying tumor growth and metastasis formation. A drug screening should be undertaken and the knowledge about the interaction of VAP-A with FFAT motif-containing protein partners, including sterol-binding proteins, would be useful to design such chemical compounds. In addition to cancers, this new pathway may be involved in the delivery of biomaterials (e.g., cell surface receptors) to the nuclear compartment as explained above, and hence other applications can be envisaged.

Although EVs do not have the ability to promote their own replication in host cells unlike enveloped viruses, numerous facets are nonetheless shared between these small phospholipid membrane-enclosed entities, including certain aspects of their biogenesis and release into the extracellular milieu (reviewed in Refs [247,248,249]). The similarity between them is further supported by discoveries revealing that virus-infected cells release modified EVs (i.e., exosomes) containing viral proteins and nucleic acids such as non-coding regulatory miRNAs [250], which could enhance or interfere with infection and/or provoke immune dysregulation [251]. These observations among others have highlighted that the exosomal and/or microvesicle pathways can be hijacked by viruses, notably retroviruses [252]. Moreover, EVs can provide an “envelope” to non-enveloped viruses such as hepatitis, which promotes their spreading without lysis of infected cells [253,254]. The latter observation might also question a “dogmatic” issue that categorizes viruses into enveloped and non-enveloped ones. The internalization of EVs and enveloped viruses might also share some similarity [255]. Direct fusion with the host cell plasma membrane or receptor-mediated endocytosis can explain the entry mechanisms of both particle types (reviewed in [256]). Once internalized into the endocytic pathway, viral components can reach the nuclear compartment, which raises the possibility that they might use the nucleoplasmic reticulum-associated late endosomes to transfer their nucleic acids to the nucleoplasm of host cells. It might be more than a coincidence that the infection of certain viruses (e.g., herpesvirus) could promote morphological alterations of the nuclear membrane of host cells and a redistribution of nuclear pore proteins, notably in NEI-like structures [257]. The interaction of viral proteins with nucleoporins might be significant in these processes [258]. Such nuclear membrane re-organization of infected cells is in line with the impact of EVs on the number of NEI [5], hence the spatial configuration of the nucleus of EV-exposed cells. Therefore, it will be of interest to revisit the cellular entry of enveloped viruses and the delivery of their viral genome into nucleus in light of the shuttling of EV cargo described here.

Lastly, the discovery of nucleoplasmic reticulum-associated late endosomes might open new avenues to deliver and target specific drugs to nuclei of cancer cells or other pathologic cells. Altogether, deciphering at a molecular level all spatiotemporal multi-steps of this new intracellular pathway would benefit the field of nanobiological technology towards new medical therapeutic approaches [91].

## Figures and Tables

**Table 1:** Potential pathways of nuclear transport of cell surface proteins present at plasma membrane and/or extracellular membrane vesicles.

Retrograde Transport	Nuclear Envelope-Associated Early Endosomes	Nuclear Envelope Invagination-Associated Late Endosomes
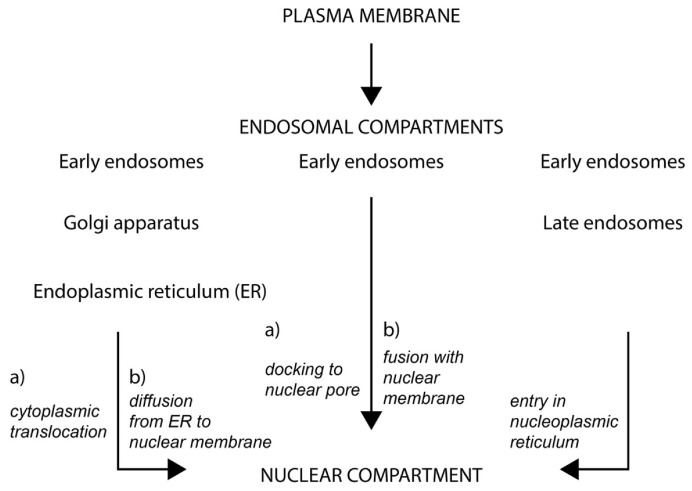		
**Implication of Nuclear Pores**		
YES	YES	YES
**Implication of Importin β1**		
YES	YES (a)/unclear (b)	YES
**Fusion of Endosome with the Outer Nuclear Membrane**		
–	NO (a)/YES (b)	Not determined
**Implication of Sec61 Translocon Complex**		
YES	unclear (a)/YES (b)	Not determined
[240,241,243,244,245,246]	[232,236,238,242,246]	[5,91,160]

a, b; two alternative routes.

## References

[1] Ratajczak J., Wysoczynski M., Hayek F., Janowska-Wieczorek A., Ratajczak M.Z. (2006). Membrane-derived microvesicles: Important and underappreciated mediators of cell-to-cell communication. Leukemia.

[2] Simons M., Raposo G. (2009). Exosomes—Vesicular carriers for intercellular communication. Current. Opin. Cell Biol..

[3] Raposo G., Stoorvogel W. (2013). Extracellular vesicles: Exosomes, microvesicles, and friends. J. Cell Biol..

[4] Tkach M., Théry C. (2016). Communication by extracellular vesicles: Where we are and where we need to go. Cell.

[5] Rappa G., Santos M.F., Green T.M., Karbanová J., Hassler J., Bai Y., Barsky S.H., Corbeil D., Lorico A. (2017). Nuclear transport of cancer extracellular vesicle-derived biomaterials through nuclear envelope invagination-associated late endosomes. Oncotarget.

[6] Read J., Ingram A., Al Saleh H.A., Platko K., Gabriel K., Kapoor A., Pinthus J., Majeed F., Qureshi T., Al-Nedawi K. (2017). Nuclear transportation of exogenous epidermal growth factor receptor and androgen receptor via extracellular vesicles. Eur. J. Cancer.

[7] Zhang H., Freitas D., Kim H.S., Fabijanic K., Li Z., Chen H., Mark M.T., Molina H., Martin A.B., Bojmar L. (2018). Identification of distinct nanoparticles and subsets of extracellular vesicles by asymmetric flow field-flow fractionation. Nat. Cell Biol..

[8] Zijlstra A., Di Vizio D. (2018). Size matters in nanoscale communication. Nat. Cell Biol..

[9] Caruso S., Poon I.K.H. (2018). Apoptotic cell-derived extracellular vesicles: More than just debris. Front. Immunol..

[10] Meehan B., Rak J., Di Vizio D. (2016). Oncosomes—Large and small: What are they, where they came from?. J. Extracell. Vesicles.

[11] Di Vizio D., Kim J., Hager M.H., Morello M., Yang W., Lafargue C.J., True L.D., Rubin M.A., Adam R.M., Beroukhim R. (2009). Oncosome formation in prostate cancer: Association with a region of frequent chromosomal deletion in metastatic disease. Cancer Res..

[12] Johnson S.M., Dempsey C., Parker C., Mironov A., Bradley H., Saha V. (2017). Acute lymphoblastic leukaemia cells produce large extracellular vesicles containing organelles and an active cytoskeleton. J. Extracell. Vesicles.

[13] Tanaka Y., Okada Y., Hirokawa N. (2005). FGF-induced vesicular release of Sonic hedgehog and retinoic acid in leftward nodal flow is critical for left-right determination. Nature.

[14] Panakova D., Sprong H., Marois E., Thiele C., Eaton S. (2005). Lipoprotein particles are required for hedgehog and wingless signalling. Nature.

[15] Ratajczak J., Miekus K., Kucia M., Zhang J., Reca R., Dvorak P., Ratajczak M.Z. (2006). Embryonic stem cell-derived microvesicles reprogram hematopoietic progenitors: Evidence for horizontal transfer of mRNA and protein delivery. Leukemia.

[16] Valadi H., Ekstrom K., Bossios A., Sjostrand M., Lee J.J., Lotvall J.O. (2007). Exosome-mediated transfer of mRNAs and microRNAs is a novel mechanism of genetic exchange between cells. Nat. Cell Biol..

[17] Ridder K., Sevko A., Heide J., Dams M., Rupp A.K., Macas J., Starmann J., Tjwa M., Plate K.H., Sultmann H. (2015). Extracellular vesicle-mediated transfer of functional RNA in the tumor microenvironment. Oncoimmunology.

[18] Ratajczak M.Z., Ratajczak J. (2016). Horizontal transfer of RNA and proteins between cells by extracellular microvesicles: 14 years later. Clin. Transl. Med..

[19] Zhang W.L., Liu Y., Jiang J., Tang Y.J., Tang Y.L., Liang X.H. (2020). Extracellular vesicle long non-coding RNA-mediated crosstalk in the tumor microenvironment: Tiny molecules, huge roles. Cancer Sci..

[20] O’Brien K., Breyne K., Ughetto S., Laurent L.C., Breakefield X.O. (2020). RNA delivery by extracellular vesicles in mammalian cells and its applications. Nat. Rev. Mol. Cell Biol..

[21] Kanada M., Bachmann M.H., Hardy J.W., Frimannson D.O., Bronsart L., Wang A., Sylvester M.D., Schmidt T.L., Kaspar R.L., Butte M.J. (2015). Differential fates of biomolecules delivered to target cells via extracellular vesicles. Proc. Natl. Acad. Sci. USA.

[22] Margolis L., Sadovsky Y. (2019). The biology of extracellular vesicles: The known unknowns. PLoS Biol..

[23] Bobrie A., Colombo M., Krumeich S., Raposo G., Thery C. (2012). Diverse subpopulations of vesicles secreted by different intracellular mechanisms are present in exosome preparations obtained by differential ultracentrifugation. J. Extracell. Vesicles.

[24] Rappa G., Mercapide J., Anzanello F., Pope R.M., Lorico A. (2013). Biochemical and biological characterization of exosomes containing prominin-1/CD133. Mol. Cancer.

[25] Kowal J., Arras G., Colombo M., Jouve M., Morath J.P., Primdal-Bengtson B., Dingli F., Loew D., Tkach M., Thery C. (2016). Proteomic comparison defines novel markers to characterize heterogeneous populations of extracellular vesicle subtypes. Proc. Natl. Acad. Sci. USA.

[26] Tkach M., Kowal J., Théry C. (2018). Why the need and how to approach the functional diversity of extracellular vesicles. Philos. Trans. R. Soc. Lond. B Biol. Sci..

[27] Théry C., Witwer K.W., Aikawa E., Alcaraz M.J., Anderson J.D., Andriantsitohaina R., Antoniou A., Arab T., Archer F., Atkin-Smith G.K. (2018). Minimal information for studies of extracellular vesicles 2018 (MISEV2018): A position statement of the international society for extracellular vesicles and update of the MISEV2014 guidelines. J. Extracell. Vesicles.

[28] Martins T.S., Trindade D., Vaz M., Campelo I., Almeida M., Trigo G., da Cruz E.S.O.A.B., Henriques A.G. (2020). Diagnostic and therapeutic potential of exosomes in Alzheimer’s disease. J. Neurochem..

[29] Nazarenko I. (2020). Extracellular vesicles: Recent developments in technology and perspectives for cancer liquid biopsy. Recent Results Cancer Res..

[30] Beit-Yannai E., Tabak S., Stamer W.D. (2018). Physical exosome: Exosome interactions. J. Cell Mol. Med..

[31] Yáñez-Mó M., Siljander P.R., Andreu Z., Zavec A.B., Borras F.E., Buzas E.I., Buzas K., Casal E., Cappello F., Carvalho J. (2015). Biological properties of extracellular vesicles and their physiological functions. J. Extracell. Vesicles.

[32] Desrochers L.M., Antonyak M.A., Cerione R.A. (2016). Extracellular vesicles: Satellites of information transfer in cancer and stem cell biology. Dev. Cell.

[33] McIver S.C., Katsumura K.R., Davids E., Liu P., Kang Y.A., Yang D., Bresnick E.H. (2016). Exosome complex orchestrates developmental signaling to balance proliferation and differentiation during erythropoiesis. eLife.

[34] Janas A.M., Sapon K., Janas T., Stowell M.H., Janas T. (2016). Exosomes and other extracellular vesicles in neural cells and neurodegenerative diseases. Biochim. Biophys. Acta.

[35] Fröhlich D., Kuo W.P., Fruhbeis C., Sun J.J., Zehendner C.M., Luhmann H.J., Pinto S., Toedling J., Trotter J., Krämer-Albers E.M. (2014). Multifaceted effects of oligodendroglial exosomes on neurons: Impact on neuronal firing rate, signal transduction and gene regulation. Philos. Trans. R. Soc. Lond. B Biol. Sci..

[36] Fruhbeis C., Frohlich D., Kuo W.P., Amphornrat J., Thilemann S., Saab A.S., Kirchhoff F., Mobius W., Goebbels S., Nave K.A. (2013). Neurotransmitter-triggered transfer of exosomes mediates oligodendrocyte-neuron communication. PLoS Biol..

[37] Rajendran L., Honsho M., Zahn T.R., Keller P., Geiger K.D., Verkade P., Simons K. (2006). Alzheimer’s disease beta-amyloid peptides are released in association with exosomes. Proc. Natl. Acad. Sci. USA.

[38] Bellingham S.A., Guo B.B., Coleman B.M., Hill A.F. (2012). Exosomes: Vehicles for the transfer of toxic proteins associated with neurodegenerative diseases?. Front. Physiol..

[39] Coleman B.M., Hill A.F. (2015). Extracellular vesicles--Their role in the packaging and spread of misfolded proteins associated with neurodegenerative diseases. Semin. Cell Dev. Biol..

[40] Skog J., Wurdinger T., van Rijn S., Meijer D.H., Gainche L., Sena-Esteves M., Curry W.T., Carter B.S., Krichevsky A.M., Breakefield X.O. (2008). Glioblastoma microvesicles transport RNA and proteins that promote tumour growth and provide diagnostic biomarkers. Nat. Cell Biol..

[41] Becker A., Thakur B.K., Weiss J.M., Kim H.S., Peinado H., Lyden D. (2016). Extracellular Vesicles in Cancer: Cell-to-Cell Mediators of Metastasis. Cancer Cell.

[42] Syn N., Wang L., Sethi G., Thiery J.P., Goh B.C. (2016). Exosome-Mediated Metastasis: From Epithelial-Mesenchymal Transition to Escape from Immunosurveillance. Trends Pharmacol. Sci..

[43] Morad G., Moses M.A. (2019). Brainwashed by extracellular vesicles: The role of extracellular vesicles in primary and metastatic brain tumour microenvironment. J. Extracell. Vesicles.

[44] Al-Nedawi K., Meehan B., Micallef J., Lhotak V., May L., Guha A., Rak J. (2008). Intercellular transfer of the oncogenic receptor EGFRvIII by microvesicles derived from tumour cells. Nat. Cell Biol..

[45] Abdouh M., Hamam D., Gao Z.H., Arena V., Arena M., Arena G.O. (2017). Exosomes isolated from cancer patients’ sera transfer malignant traits and confer the same phenotype of primary tumors to oncosuppressor-mutated cells. J. Exp. Clin. Cancer Res..

[46] Costa-Silva B., Aiello N.M., Ocean A.J., Singh S., Zhang H., Thakur B.K., Becker A., Hoshino A., Mark M.T., Molina H. (2015). Pancreatic cancer exosomes initiate pre-metastatic niche formation in the liver. Nat. Cell Biol..

[47] Peinado H., Aleckovic M., Lavotshkin S., Matei I., Costa-Silva B., Moreno-Bueno G., Hergueta-Redondo M., Williams C., Garcia-Santos G., Ghajar C. (2012). Melanoma exosomes educate bone marrow progenitor cells toward a pro-metastatic phenotype through MET. Nat. Med..

[48] Corbeil D., Loriro A., Edelstein L., Smythies J., Quesenberry P., Noble D. (2020). Exosomes, microvesicles, and their friends in solid tumors. Exosomes: A Clinical Compendium.

[49] Kowal J., Tkach M., Théry C. (2014). Biogenesis and secretion of exosomes. Curr. Opin. Cell Biol..

[50] Corbeil D., Röper K., Fargeas C.A., Joester A., Huttner W.B. (2001). Prominin: A story of cholesterol, plasma membrane protrusions and human pathology. Traffic.

[51] Thamm K., Šimaité D., Karbanová J., Bermúdez V., Reichert D., Morgenstern A., Bornhäuser M., Huttner W.B., Wilsch-Bräuninger M., Corbeil D. (2019). Prominin-1 (CD133) modulates the architecture and dynamics of microvilli. Traffic.

[52] Singer D., Thamm K., Zhuang H., Karbanová J., Gao Y., Walker J.V., Jin H., Wu X., Coveney C.R., Marangoni P. (2019). Prominin-1 controls stem cell activation by orchestrating ciliary dynamics. EMBO J..

[53] Marzesco A.M. (2013). Prominin-1-containing membrane vesicles: Origins, formation, and utility. Adv. Exp. Med. Biol..

[54] Bauer N., Wilsch-Bräuninger M., Karbanová J., Fonseca A.V., Strauss D., Freund D., Thiele C., Huttner W.B., Bornhäuser M., Corbeil D. (2011). Haematopoietic stem cell differentiation promotes the release of prominin-1/CD133-containing membrane vesicles—A role of the endocytic-exocytic pathway. EMBO Mol. Med..

[55] Karbanová J., Laco J., Marzesco A.M., Janich P., Vobornikova M., Mokry J., Fargeas C.A., Huttner W.B., Corbeil D. (2014). Human prominin-1 (CD133) is detected in both neoplastic and non-neoplastic salivary gland diseases and released into saliva in a ubiquitinated form. PLoS ONE.

[56] Yang F., Xing Y., Li Y., Chen X., Jiang J., Ai Z., Wei Y. (2018). Monoubiquitination of cancer stem cell marker CD133 at lysine 848 regulates its secretion and promotes cell migration. Mol. Cell Biol..

[57] Baietti M.F., Zhang Z., Mortier E., Melchior A., Degeest G., Geeraerts A., Ivarsson Y., Depoortere F., Coomans C., Vermeiren E. (2012). Syndecan-syntenin-ALIX regulates the biogenesis of exosomes. Nat. Cell Biol..

[58] Raiborg C., Stenmark H. (2009). The ESCRT machinery in endosomal sorting of ubiquitylated membrane proteins. Nature.

[59] Colombo M., Raposo G., Théry C. (2014). Biogenesis, secretion, and intercellular interactions of exosomes and other extracellular vesicles. Annu. Rev. Cell Dev. Biol..

[60] Van Niel G., D’Angelo G., Raposo G. (2018). Shedding light on the cell biology of extracellular vesicles. Nat. Rev. Mol. Cell Biol..

[61] Dubreuil V., Marzesco A.M., Corbeil D., Huttner W.B., Wilsch-Bräuninger M. (2007). Midbody and primary cilium of neural progenitors release extracellular membrane particles enriched in the stem cell marker prominin-1. J. Cell Biol..

[62] Marzesco A.M., Janich P., Wilsch-Bräuninger M., Dubreuil V., Langenfeld K., Corbeil D., Huttner W.B. (2005). Release of extracellular membrane particles carrying the stem cell marker prominin-1 (CD133) from neural progenitors and other epithelial cells. J. Cell Sci..

[63] Nager A.R., Goldstein J.S., Herranz-Perez V., Portran D., Ye F., Garcia-Verdugo J.M., Nachury M.V. (2017). An actin network dispatches ciliary GPCRs into extracellular vesicles to modulate signaling. Cell.

[64] Kang M., Kim S., Ko J. (2019). Roles of CD133 in microvesicle formation and oncoprotein trafficking in colon cancer. FASEB J..

[65] Wehman A.M., Poggioli C., Schweinsberg P., Grant B.D., Nance J. (2011). The P4-ATPase TAT-5 inhibits the budding of extracellular vesicles in C. elegans embryos. Curr Biol..

[66] Ettinger A.W., Wilsch-Bräuninger M., Marzesco A.M., Bickle M., Lohmann A., Maliga Z., Karbanová J., Corbeil D., Hyman A.A., Huttner W.B. (2011). Proliferating versus differentiating stem and cancer cells exhibit distinct midbody-release behaviour. Nat. Commun..

[67] Trajkovic K., Hsu C., Chiantia S., Rajendran L., Wenzel D., Wieland F., Schwille P., Brügger B., Simons M. (2008). Ceramide triggers budding of exosome vesicles into multivesicular endosomes. Science.

[68] Marzesco A.M., Wilsch-Bräuninger M., Dubreuil V., Janich P., Langenfeld K., Thiele C., Huttner W.B., Corbeil D. (2009). Release of extracellular membrane vesicles from microvilli of epithelial cells is enhanced by depleting membrane cholesterol. FEBS Lett..

[69] Skotland T., Hessvik N.P., Sandvig K., Llorente A. (2019). Exosomal lipid composition and the role of ether lipids and phosphoinositides in exosome biology. J. Lipid Res..

[70] Zöller M. (2009). Tetraspanins: Push and pull in suppressing and promoting metastasis. Nat. Rev. Cancer.

[71] Andreu Z., Yáñez-Mó M. (2014). Tetraspanins in extracellular vesicle formation and function. Front. Immunol..

[72] Wang Z., Zöller M. (2019). Exosomes, metastases, and the miracle of cancer stem cell markers. Cancer Met. Rev..

[73] Wartlick O., Kicheva A., Gonzalez-Gaitan M. (2009). Morphogen gradient formation. Cold Spring Harb. Perspect. Biol..

[74] Yu S.R., Burkhardt M., Nowak M., Ries J., Petrasek Z., Scholpp S., Schwille P., Brand M. (2009). Fgf8 morphogen gradient forms by a source-sink mechanism with freely diffusing molecules. Nature.

[75] Hoshino A., Costa-Silva B., Shen T.L., Rodrigues G., Hashimoto A., Tesic Mark M., Molina H., Kohsaka S., Di Giannatale A., Ceder S. (2015). Tumour exosome integrins determine organotropic metastasis. Nature.

[76] Hurwitz S.N., Meckes D.G. (2019). Extracellular vesicle integrins distinguish unique cancers. Proteomes.

[77] Lu H., Bowler N., Harshyne L.A., Craig Hooper D., Krishn S.R., Kurtoglu S., Fedele C., Liu Q., Tang H.Y., Kossenkov A.V. (2018). Exosomal alphavbeta6 integrin is required for monocyte M2 polarization in prostate cancer. Matrix Biol..

[78] DeRita R.M., Sayeed A., Garcia V., Krishn S.R., Shields C.D., Sarker S., Friedman A., McCue P., Molugu S.K., Rodeck U. (2019). Tumor-derived extracellular vesicles require beta1 integrins to promote anchorage-independent growth. iScience.

[79] Hazan-Halevy I., Rosenblum D., Weinstein S., Bairey O., Raanani P., Peer D. (2015). Cell-specific uptake of mantle cell lymphoma-derived exosomes by malignant and non-malignant B-lymphocytes. Cancer Lett..

[80] Hogan M.C., Manganelli L., Woollard J.R., Masyuk A.I., Masyuk T.V., Tammachote R., Huang B.Q., Leontovich A.A., Beito T.G., Madden B.J. (2009). Characterization of PKD protein-positive exosome-like vesicles. J. Am. Soc. Nephrol..

[81] Heusermann W., Hean J., Trojer D., Steib E., von Bueren S., Graff-Meyer A., Genoud C., Martin K., Pizzato N., Voshol J. (2016). Exosomes surf on filopodia to enter cells at endocytic hot spots, traffic within endosomes, and are targeted to the ER. J. Cell Biol..

[82] Prada I., Meldolesi J. (2016). Binding and fusion of extracellular vesicles to the plasma membrane of their cell targets. Int. J. Mol. Sci..

[83] French K.C., Antonyak M.A., Cerione R.A. (2017). Extracellular vesicle docking at the cellular port: Extracellular vesicle binding and uptake. Semin. Cell Dev. Biol..

[84] Bruno S., Grange C., Deregibus M.C., Calogero R.A., Saviozzi S., Collino F., Morando L., Busca A., Falda M., Bussolati B. (2009). Mesenchymal stem cell-derived microvesicles protect against acute tubular injury. J. Am. Soc. Nephrol..

[85] Barres C., Blanc L., Bette-Bobillo P., Andre S., Mamoun R., Gabius H.J., Vidal M. (2010). Galectin-5 is bound onto the surface of rat reticulocyte exosomes and modulates vesicle uptake by macrophages. Blood.

[86] Paolillo M., Schinelli S. (2017). Integrins and exosomes, a dangerous liaison in cancer progression. Cancers.

[87] Morelli A.E., Larregina A.T., Shufesky W.J., Sullivan M.L., Stolz D.B., Papworth G.D., Zahorchak A.F., Logar A.J., Wang Z., Watkins S.C. (2004). Endocytosis, intracellular sorting, and processing of exosomes by dendritic cells. Blood.

[88] Rubartelli A., Poggi A., Zocchi M.R. (1997). The selective engulfment of apoptotic bodies by dendritic cells is mediated by the alpha(v)beta3 integrin and requires intracellular and extracellular calcium. Eur. J. Immunol..

[89] Fitzner D., Schnaars M., van Rossum D., Krishnamoorthy G., Dibaj P., Bakhti M., Regen T., Hanisch U.K., Simons M. (2011). Selective transfer of exosomes from oligodendrocytes to microglia by macropinocytosis. J. Cell Sci..

[90] Moller-Tank S., Maury W. (2014). Phosphatidylserine receptors: Enhancers of enveloped virus entry and infection. Virology.

[91] Santos M.F., Rappa G., Karbanová J., Vanier C., Morimoto C., Corbeil D., Lorico A. (2019). Anti-human CD9 antibody Fab fragment impairs the internalization of extracellular vesicles and the nuclear transfer of their cargo proteins. J. Cell Mol. Med..

[92] Tian Y., Li S., Song J., Ji T., Zhu M., Anderson G.J., Wei J., Nie G. (2014). A doxorubicin delivery platform using engineered natural membrane vesicle exosomes for targeted tumor therapy. Biomaterials.

[93] Nazimek K., Bryniarski K. (2020). Perspectives in manipulating EVs for therapeutic applications: Focus on cancer treatment. Int. J. Mol. Sci..

[94] Zhao X., Wu D., Ma X., Wang J., Hou W., Zhang W. (2020). Exosomes as drug carriers for cancer therapy and challenges regarding exosome uptake. Biomed. Pharmacother..

[95] Mulcahy L.A., Pink R.C., Carter D.R. (2014). Routes and mechanisms of extracellular vesicle uptake. J. Extracell. Vesicles.

[96] McKelvey K.J., Powell K.L., Ashton A.W., Morris J.M., McCracken S.A. (2015). Exosomes: Mechanisms of uptake. J. Circ. Biomark..

[97] Mathieu M., Martin-Jaular L., Lavieu G., Théry C. (2019). Specificities of secretion and uptake of exosomes and other extracellular vesicles for cell-to-cell communication. Nat. Cell Biol..

[98] Raposo G., Nijman H.W., Stoorvogel W., Liejendekker R., Harding C.V., Melief C.J., Geuze H.J. (1996). B lymphocytes secrete antigen-presenting vesicles. J. Exp. Med..

[99] Zitvogel L., Regnault A., Lozier A., Wolfers J., Flament C., Tenza D., Ricciardi-Castagnoli P., Raposo G., Amigorena S. (1998). Eradication of established murine tumors using a novel cell-free vaccine: Dendritic cell-derived exosomes. Nat. Med..

[100] Hakulinen J., Junnikkala S., Sorsa T., Meri S. (2004). Complement inhibitor membrane cofactor protein (MCP.; CD46) is constitutively shed from cancer cell membranes in vesicles and converted by a metalloproteinase to a functionally active soluble form. Eur. J. Immunol..

[101] Tkach M., Kowal J., Zucchetti A.E., Enserink L., Jouve M., Lankar D., Saitakis M., Martin-Jaular L., Théry C. (2017). Qualitative differences in T-cell activation by dendritic cell-derived extracellular vesicle subtypes. EMBO J..

[102] Montecalvo A., Larregina A.T., Shufesky W.J., Stolz D.B., Sullivan M.L., Karlsson J.M., Baty C.J., Gibson G.A., Erdos G., Wang Z. (2012). Mechanism of transfer of functional microRNAs between mouse dendritic cells via exosomes. Blood.

[103] Parolini I., Federici C., Raggi C., Lugini L., Palleschi S., De Milito A., Coscia C., Iessi E., Logozzi M., Molinari A. (2009). Microenvironmental pH is a key factor for exosome traffic in tumor cells. J. Biol. Chem..

[104] Mothes W., Boerger A.L., Narayan S., Cunningham J.M., Young J.A. (2000). Retroviral entry mediated by receptor priming and low pH triggering of an envelope glycoprotein. Cell.

[105] Bonsergent E., Lavieu G. (2019). Content release of extracellular vesicles in a cell-free extract. FEBS Lett..

[106] Nour A.M., Modis Y. (2014). Endosomal vesicles as vehicles for viral genomes. Trends Cell Biol..

[107] Hemler M.E. (2003). Tetraspanin proteins mediate cellular penetration, invasion, and fusion events and define a novel type of membrane microdomain. Annu. Rev. Cell Dev. Biol..

[108] Del Conde I., Shrimpton C.N., Thiagarajan P., Lopez J.A. (2005). Tissue-factor-bearing microvesicles arise from lipid rafts and fuse with activated platelets to initiate coagulation. Blood.

[109] Gonda A., Kabagwira J., Senthil G.N., Wall N.R. (2019). Internalization of exosomes through receptor-mediated endocytosis. Mol. Cancer Res..

[110] Delenclos M., Trendafilova T., Mahesh D., Baine A.M., Moussaud S., Yan I.K., Patel T., McLean P.J. (2017). Investigation of endocytic pathways for the internalization of exosome-associated oligomeric Alpha-Synuclein. Front. Neurosci..

[111] Tian T., Wang Y., Wang H., Zhu Z., Xiao Z. (2010). Visualizing of the cellular uptake and intracellular trafficking of exosomes by live-cell microscopy. J. Cell Biochem..

[112] Christianson H.C., Svensson K.J., Belting M. (2014). Exosome and microvesicle mediated phene transfer in mammalian cells. Semin. Cancer Biol..

[113] Meldolesi J. (2018). Exosomes and ectosomes in intercellular communication. Curr. Biol..

[114] Tian T., Zhu Y.L., Zhou Y.Y., Liang G.F., Wang Y.Y., Hu F.H., Xiao Z.D. (2014). Exosome uptake through clathrin-mediated endocytosis and macropinocytosis and mediating miR-21 delivery. J. Biol. Chem..

[115] Nanbo A., Kawanishi E., Yoshida R., Yoshiyama H. (2013). Exosomes derived from Epstein-Barr virus-infected cells are internalized via caveola-dependent endocytosis and promote phenotypic modulation in target cells. J. Virol..

[116] Rai A.K., Johnson P.J. (2019). Trichomonas vaginalis extracellular vesicles are internalized by host cells using proteoglycans and caveolin-dependent endocytosis. Proc. Natl. Acad. Sci. USA.

[117] Svensson K.J., Christianson H.C., Wittrup A., Bourseau-Guilmain E., Lindqvist E., Svensson L.M., Morgelin M., Belting M. (2013). Exosome uptake depends on ERK1/2-heat shock protein 27 signaling and lipid Raft-mediated endocytosis negatively regulated by caveolin-1. J. Biol. Chem..

[118] Plebanek M.P., Mutharasan R.K., Volpert O., Matov A., Gatlin J.C., Thaxton C.S. (2015). Nanoparticle targeting and cholesterol flux through scavenger receptor type B-1 inhibits cellular exosome uptake. Sci. Rep..

[119] Subtil A., Hemar A., Dautry-Varsat A. (1994). Rapid endocytosis of interleukin 2 receptors when clathrin-coated pit endocytosis is inhibited. J. Cell Sci..

[120] Hayer A., Stoeber M., Ritz D., Engel S., Meyer H.H., Helenius A. (2010). Caveolin-1 is ubiquitinated and targeted to intralumenal vesicles in endolysosomes for degradation. J. Cell Biol..

[121] Pelkmans L., Helenius A. (2002). Endocytosis via caveolae. Traffic.

[122] Parton R.G., Howes M.T. (2010). Revisiting caveolin trafficking: The end of the caveosome. J. Cell Biol..

[123] Mayor S., Parton R.G., Donaldson J.G. (2014). Clathrin-independent pathways of endocytosis. Cold Spring Harb. Perspect. Biol..

[124] Chaudhary N., Gomez G.A., Howes M.T., Lo H.P., McMahon K.A., Rae J.A., Schieber N.L., Hill M.M., Gaus K., Yap A.S. (2014). Endocytic crosstalk: Cavins, caveolins, and caveolae regulate clathrin-independent endocytosis. PLoS Biol..

[125] Kovtun O., Tillu V.A., Ariotti N., Parton R.G., Collins B.M. (2015). Cavin family proteins and the assembly of caveolae. J. Cell Sci..

[126] Henley J.R., Krueger E.W., Oswald B.J., McNiven M.A. (1998). Dynamin-mediated internalization of caveolae. J. Cell Biol..

[127] Rothberg K.G., Heuser J.E., Donzell W.C., Ying Y.S., Glenney J.R., Anderson R.G. (1992). Caveolin, a protein component of caveolae membrane coats. Cell.

[128] Lamaze C., Dujeancourt A., Baba T., Lo C.G., Benmerah A., Dautry-Varsat A. (2001). Interleukin 2 receptors and detergent-resistant membrane domains define a clathrin-independent endocytic pathway. Mol. Cell.

[129] Otto G.P., Nichols B.J. (2011). The roles of flotillin microdomains--endocytosis and beyond. J. Cell Sci..

[130] Koumangoye R.B., Sakwe A.M., Goodwin J.S., Patel T., Ochieng J. (2011). Detachment of breast tumor cells induces rapid secretion of exosomes which subsequently mediate cellular adhesion and spreading. PLoS ONE.

[131] Nakase I., Kobayashi N.B., Takatani-Nakase T., Yoshida T. (2015). Active macropinocytosis induction by stimulation of epidermal growth factor receptor and oncogenic Ras expression potentiates cellular uptake efficacy of exosomes. Sci. Rep..

[132] Costa Verdera H., Gitz-Francois J.J., Schiffelers R.M., Vader P. (2017). Cellular uptake of extracellular vesicles is mediated by clathrin-independent endocytosis and macropinocytosis. J. Control. Release.

[133] Feng D., Zhao W.L., Ye Y.Y., Bai X.C., Liu R.Q., Chang L.F., Zhou Q., Sui S.F. (2010). Cellular internalization of exosomes occurs through phagocytosis. Traffic.

[134] Luga V., Wrana J.L. (2013). Tumor-stroma interaction: Revealing fibroblast-secreted exosomes as potent regulators of Wnt-planar cell polarity signaling in cancer metastasis. Cancer Res..

[135] Vanlandingham P.A., Ceresa B.P. (2009). Rab7 regulates late endocytic trafficking downstream of multivesicular body biogenesis and cargo sequestration. J. Biol. Chem..

[136] Xu J., Camfield R., Gorski S.M. (2018). The interplay between exosomes and autophagy—Partners in crime. J. Cell Sci..

[137] Hurwitz S.N., Cheerathodi M.R., Nkosi D., York S.B., Meckes D.G.J. (2018). Tetraspanin CD63 bridges autophagic and endosomal processes to regulate exosomal secretion and intracellular signaling of epstein-barr virus LMP1. J. Virol..

[138] Bissig C., Gruenberg J. (2014). ALIX and the multivesicular endosome: ALIX in Wonderland. Trends Cell Biol..

[139] Record M., Silvente-Poirot S., Poirot M., Wakelam M.J.O. (2018). Extracellular vesicles: Lipids as key components of their biogenesis and functions. J. Lipid Res..

[140] Rocha N., Kuijl C., van der Kant R., Janssen L., Houben D., Janssen H., Zwart W., Neefjes J. (2009). Cholesterol sensor ORP1L contacts the ER protein VAP to control Rab7-RILP-p150 Glued and late endosome positioning. J. Cell Biol..

[141] Lui P.P., Kong S.K., Kwok T.T., Lee C.Y. (1998). The nucleus of HeLa cell contains tubular structures for Ca^2+^ signalling. Biochem. Biophys Res. Commun..

[142] Echevarria W., Leite M.F., Guerra M.T., Zipfel W.R., Nathanson M.H. (2003). Regulation of calcium signals in the nucleus by a nucleoplasmic reticulum. Nat. Cell Biol..

[143] Bootman M.D., Fearnley C., Smyrnias I., MacDonald F., Roderick H.L. (2009). An update on nuclear calcium signalling. J. Cell Sci..

[144] Prunuske A.J., Ullman K.S. (2006). The nuclear envelope: Form and reformation. Curr. Opin. Cell Biol..

[145] Malhas A., Goulbourne C., Vaux D.J. (2011). The nucleoplasmic reticulum: Form and function. Trends Cell Biol..

[146] Jorgens D.M., Inman J.L., Wojcik M., Robertson C., Palsdottir H., Tsai W.T., Huang H., Bruni-Cardoso A., Lopez C.S., Bissell M.J. (2017). Deep nuclear invaginations are linked to cytoskeletal filaments—Integrated bioimaging of epithelial cells in 3D culture. J. Cell Sci..

[147] Goulbourne C.N., Malhas A.N., Vaux D.J. (2011). The induction of a nucleoplasmic reticulum by prelamin a accumulation requires CTP: Phosphocholine cytidylyltransferase-alpha. J. Cell Sci..

[148] Storch K.N., Taatjes D.J., Bouffard N.A., Locknar S., Bishop N.M., Langevin H.M. (2007). Alpha smooth muscle actin distribution in cytoplasm and nuclear invaginations of connective tissue fibroblasts. Histochem. Cell Biol..

[149] Johnson N., Krebs M., Boudreau R., Giorgi G., LeGros M., Larabell C. (2003). Actin-filled nuclear invaginations indicate degree of cell de-differentiation. Differentiation.

[150] Saltel F., Giese A., Azzi L., Elatmani H., Costet P., Ezzoukhry Z., Dugot-Senant N., Miquerol L., Boussadia O., Wodrich H. (2017). Unr defines a novel class of nucleoplasmic reticulum involved in mRNA translation. J. Cell Sci..

[151] Fricker M., Hollinshead M., White N., Vaux D. (1997). The convoluted nucleus. Trends Cell Biol..

[152] Drozdz M.M., Jiang H., Pytowski L., Grovenor C., Vaux D.J. (2017). Formation of a nucleoplasmic reticulum requires de novo assembly of nascent phospholipids and shows preferential incorporation of nascent lamins. Sci. Rep..

[153] Haider A., Wei Y.C., Lim K., Barbosa A.D., Liu C.H., Weber U., Mlodzik M., Oras K., Collier S., Hussain M.M. (2018). PCYT1A regulates phosphatidylcholine homeostasis from the inner nuclear membrane in response to membrane stored curvature elastic stress. Dev. Cell.

[154] Bozelli J.C.J., Jennings W., Black S., Hou Y.H., Lameire D., Chatha P., Kimura T., Berno B., Khondker A., Rheinstadter M.C. (2018). Membrane curvature allosterically regulates the phosphatidylinositol cycle, controlling its rate and acyl-chain composition of its lipid intermediates. J. Biol. Chem..

[155] Marelli M., Lusk C.P., Chan H., Aitchison J.D., Wozniak R.W. (2001). A link between the synthesis of nucleoporins and the biogenesis of the nuclear envelope. J. Cell Biol..

[156] Drozdz M.M., Vaux D.J. (2017). Shared mechanisms in physiological and pathological nucleoplasmic reticulum formation. Nucleus.

[157] Pombo A., Dillon N. (2015). Three-dimensional genome architecture: Players and mechanisms. Nat. Rev. Mol. Cell Biol..

[158] Bussolati G., Marchio C., Gaetano L., Lupo R., Sapino A. (2008). Pleomorphism of the nuclear envelope in breast cancer: A new approach to an old problem. J. Cell Mol. Med..

[159] Malhas A.N., Vaux D.J. (2014). Nuclear envelope invaginations and cancer. Adv. Exp. Med. Biol..

[160] Santos M.F., Rappa G., Karbanová J., Kurth T., Corbeil D., Lorico A. (2018). VAMP-associated protein-A and oxysterol-binding protein-related protein 3 promote the entry of late endosomes into the nucleoplasmic reticulum. J. Biol. Chem..

[161] Lui P.P., Chan F.L., Suen Y.K., Kwok T.T., Kong S.K. (2003). The nucleus of HeLa cells contains tubular structures for Ca^2+^ signaling with the involvement of mitochondria. Biochem. Biophys. Res. Commun..

[162] Van der Kant R., Zondervan I., Janssen L., Neefjes J. (2013). Cholesterol-binding molecules MLN64 and ORP1L mark distinct late endosomes with transporters ABCA3 and NPC1. J. Lipid Res..

[163] Wilhelm L.P., Wendling C., Vedie B., Kobayashi T., Chenard M.P., Tomasetto C., Drin G., Alpy F. (2017). STARD3 mediates endoplasmic reticulum-to-endosome cholesterol transport at membrane contact sites. EMBO J..

[164] Zhang M., Liu P., Dwyer N.K., Christenson L.K., Fujimoto T., Martinez F., Comly M., Hanover J.A., Blanchette-Mackie E.J., Strauss J.F. (2002). MLN64 mediates mobilization of lysosomal cholesterol to steroidogenic mitochondria. J. Biol. Chem..

[165] Charman M., Kennedy B.E., Osborne N., Karten B. (2010). MLN64 mediates egress of cholesterol from endosomes to mitochondria in the absence of functional Niemann-Pick Type C1 protein. J. Lipid Res..

[166] Van der Kant R., Fish A., Janssen L., Janssen H., Krom S., Ho N., Brummelkamp T., Carette J., Rocha N., Neefjes J. (2013). Late endosomal transport and tethering are coupled processes controlled by RILP and the cholesterol sensor ORP1L. J. Cell Sci..

[167] Lee R.K., Lui P.P., Ngan E.K., Lui J.C., Suen Y.K., Chan F., Kong S.K. (2006). The nuclear tubular invaginations are dynamic structures inside the nucleus of HeLa cells. Can. J. Physiol. Pharmacol..

[168] Lee S.H., Hadipour-Lakmehsari S., Miyake T., Gramolini A.O. (2018). Three-dimensional imaging reveals endo(sarco)plasmic reticulum-containing invaginations within the nucleoplasm of muscle. Am. J. Physiol. Cell Physiol..

[169] Saita S., Shirane M., Natume T., Iemura S., Nakayama K.I. (2009). Promotion of neurite extension by protrudin requires its interaction with vesicle-associated membrane protein-associated protein. J. Biol. Chem..

[170] Raiborg C., Wenzel E.M., Pedersen N.M., Olsvik H., Schink K.O., Schultz S.W., Vietri M., Nisi V., Bucci C., Brech A. (2015). Repeated ER-endosome contacts promote endosome translocation and neurite outgrowth. Nature.

[171] Pankiv S., Lamark T., Bruun J.A., Overvatn A., Bjorkoy G., Johansen T. (2010). Nucleocytoplasmic shuttling of p62/SQSTM1 and its role in recruitment of nuclear polyubiquitinated proteins to promyelocytic leukemia bodies. J. Biol. Chem..

[172] Cantalupo G., Alifano P., Roberti V., Bruni C.B., Bucci C. (2001). Rab-interacting lysosomal protein (RILP): The Rab7 effector required for transport to lysosomes. EMBO J..

[173] Olkkonen V.M. (2015). OSBP-related protein family in lipid transport over membrane contact sites. Lipid Insights.

[174] Goldberg M.W. (2017). Nuclear pore complex tethers to the cytoskeleton. Semin. Cell Dev. Biol..

[175] Padmakumar V.C., Libotte T., Lu W., Zaim H., Abraham S., Noegel A.A., Gotzmann J., Foisner R., Karakesisoglou I. (2005). The inner nuclear membrane protein Sun1 mediates the anchorage of Nesprin-2 to the nuclear envelope. J. Cell Sci..

[176] Banerjee I., Zhang J., Moore-Morris T., Pfeiffer E., Buchholz K.S., Liu A., Ouyang K., Stroud M.J., Gerace L., Evans S.M. (2014). Targeted ablation of nesprin 1 and nesprin 2 from murine myocardium results in cardiomyopathy, altered nuclear morphology and inhibition of the biomechanical gene response. PLoS Genet..

[177] Zhang X., Lei K., Yuan X., Wu X., Zhuang Y., Xu T., Xu R., Han M. (2009). SUN1/2 and Syne/Nesprin-1/2 complexes connect centrosome to the nucleus during neurogenesis and neuronal migration in mice. Neuron.

[178] Lei K., Zhang X., Ding X., Guo X., Chen M., Zhu B., Xu T., Zhuang Y., Xu R., Han M. (2009). SUN1 and SUN2 play critical but partially redundant roles in anchoring nuclei in skeletal muscle cells in mice. Proc. Natl. Acad. Sci. USA.

[179] Wilson M.H., Holzbaur E.L. (2015). Nesprins anchor kinesin-1 motors to the nucleus to drive nuclear distribution in muscle cells. Development.

[180] Zhou C., Rao L., Shanahan C.M., Zhang Q. (2018). Nesprin-1/2: Roles in nuclear envelope organisation, myogenesis and muscle disease. Biochem. Soc. Trans..

[181] Cai Y., Singh B.B., Aslanukov A., Zhao H., Ferreira P.A. (2001). The docking of kinesins, KIF5B and KIF5C, to Ran-binding protein 2 (RanBP2) is mediated via a novel RanBP2 domain. J. Biol. Chem..

[182] Cohen S., Valm A.M., Lippincott-Schwartz J. (2018). Interacting organelles. Curr. Opin. Cell Biol..

[183] Wu H., Carvalho P., Voeltz G.K. (2018). Here, there, and everywhere: The importance of ER membrane contact sites. Science.

[184] Wilfling F., Wang H., Haas J.T., Krahmer N., Gould T.J., Uchida A., Cheng J.X., Graham M., Christiano R., Frohlich F. (2013). Triacylglycerol synthesis enzymes mediate lipid droplet growth by relocalizing from the ER to lipid droplets. Dev. Cell.

[185] Lahiri S., Chao J.T., Tavassoli S., Wong A.K., Choudhary V., Young B.P., Loewen C.J., Prinz W.A. (2014). A conserved endoplasmic reticulum membrane protein complex (EMC) facilitates phospholipid transfer from the ER to mitochondria. PLoS Biol..

[186] Eisenberg-Bord M., Shai N., Schuldiner M., Bohnert M. (2016). A Tether is a tether is a tether: Tethering at membrane contact sites. Dev. Cell.

[187] Phillips M.J., Voeltz G.K. (2016). Structure and function of ER membrane contact sites with other organelles. Nat. Rev. Mol. Cell Biol..

[188] Eden E.R. (2016). The formation and function of ER-endosome membrane contact sites. Biochim. Biophys. Acta.

[189] Scorrano L., De Matteis M.A., Emr S., Giordano F., Hajnoczky G., Kornmann B., Lackner L.L., Levine T.P., Pellegrini L., Reinisch K. (2019). Coming together to define membrane contact sites. Nature Commun..

[190] Di Mattia T., Tomasetto C., Alpy F. (2020). Faraway, so close! Functions of endoplasmic reticulum-endosome contacts. Biochim. Biophys. Acta Mol. Cell Biol. Lipids.

[191] Alpy F., Rousseau A., Schwab Y., Legueux F., Stoll I., Wendling C., Spiegelhalter C., Kessler P., Mathelin C., Rio M.C. (2013). STARD3 or STARD3NL and VAP form a novel molecular tether between late endosomes and the ER. J. Cell Sci..

[192] Antonny B., Bigay J., Mesmin B. (2018). The oxysterol-binding protein cycle: Burning off PI(4)P to transport cholesterol. Annu. Rev. Biochem..

[193] Van der Kant R., Neefjes J. (2014). Small regulators, major consequences—Ca(2)(+) and cholesterol at the endosome-ER interface. J. Cell Sci..

[194] Peretti D., Dahan N., Shimoni E., Hirschberg K., Lev S. (2008). Coordinated lipid transfer between the endoplasmic reticulum and the Golgi complex requires the VAP proteins and is essential for Golgi-mediated transport. Mol. Biol. Cell.

[195] Wyles J.P., McMaster C.R., Ridgway N.D. (2002). Vesicle-associated membrane protein-associated protein-A (VAP-A) interacts with the oxysterol-binding protein to modify export from the endoplasmic reticulum. J. Biol. Chem..

[196] Amarilio R., Ramachandran S., Sabanay H., Lev S. (2005). Differential regulation of endoplasmic reticulum structure through VAP-Nir protein interaction. J. Biol. Chem..

[197] Kawano M., Kumagai K., Nishijima M., Hanada K. (2006). Efficient trafficking of ceramide from the endoplasmic reticulum to the Golgi apparatus requires a VAMP-associated protein-interacting FFAT motif of CERT. J. Biol. Chem..

[198] Hanada K. (2006). Discovery of the molecular machinery CERT for endoplasmic reticulum-to-Golgi trafficking of ceramide. Mol. Cell Biochem..

[199] Feng J., He L., Li Y., Xiao F., Hu G. (2018). Modeling of PH Domains and Phosphoinositides Interactions and Beyond. Protein Reviews—Purinergic Receptors.

[200] Kaiser S.E., Brickner J.H., Reilein A.R., Fenn T.D., Walter P., Brunger A.T. (2005). Structural basis of FFAT motif-mediated ER targeting. Structure.

[201] Ngo M., Ridgway N.D. (2009). Oxysterol binding protein-related Protein 9 (ORP9) is a cholesterol transfer protein that regulates Golgi structure and function. Mol. Biol. Cell.

[202] De Vos K.J., Morotz G.M., Stoica R., Tudor E.L., Lau K.F., Ackerley S., Warley A., Shaw C.E., Miller C.C. (2012). VAPB interacts with the mitochondrial protein PTPIP51 to regulate calcium homeostasis. Hum. Mol. Genet..

[203] Stoica R., De Vos K.J., Paillusson S., Mueller S., Sancho R.M., Lau K.F., Vizcay-Barrena G., Lin W.L., Xu Y.F., Lewis J. (2014). ER-mitochondria associations are regulated by the VAPB-PTPIP51 interaction and are disrupted by ALS/FTD-associated TDP-43. Nat. Commun..

[204] Manford A.G., Stefan C.J., Yuan H.L., Macgurn J.A., Emr S.D. (2012). ER-to-plasma membrane tethering proteins regulate cell signaling and ER morphology. Dev. Cell.

[205] Saheki Y., De Camilli P. (2017). Endoplasmic reticulum-plasma membrane contact sites. Annu. Rev. Biochem..

[206] Lehto M., Hynynen R., Karjalainen K., Kuismanen E., Hyvarinen K., Olkkonen V.M. (2005). Targeting of OSBP-related protein 3 (ORP3) to endoplasmic reticulum and plasma membrane is controlled by multiple determinants. Exp. Cell Res..

[207] Lehto M., Mayranpaa M.I., Pellinen T., Ihalmo P., Lehtonen S., Kovanen P.T., Groop P.H., Ivaska J., Olkkonen V.M. (2008). The R-Ras interaction partner ORP3 regulates cell adhesion. J. Cell Sci..

[208] Weber-Boyvat M., Kentala H., Lilja J., Vihervaara T., Hanninen R., Zhou Y., Peranen J., Nyman T.A., Ivaska J., Olkkonen V.M. (2015). OSBP-related protein 3 (ORP3) coupling with VAMP-associated protein A regulates R-Ras activity. Exp. Cell Res..

[209] D’Souza R.S., Lim J.Y., Turgut A., Servage K., Zhang J., Orth K., Sosale N.G., Lazzara M.J., Allegood J., Casanova J.E. (2020). Calcium-stimulated disassembly of focal adhesions mediated by an ORP3/IQSec1 complex. Elife.

[210] Suchanek M., Hynynen R., Wohlfahrt G., Lehto M., Johansson M., Saarinen H., Radzikowska A., Thiele C., Olkkonen V.M. (2007). The mammalian oxysterol-binding protein-related proteins (ORPs) bind 25-hydroxycholesterol in an evolutionarily conserved pocket. Biochem. J..

[211] Friedman J.R., Dibenedetto J.R., West M., Rowland A.A., Voeltz G.K. (2013). Endoplasmic reticulum-endosome contact increases as endosomes traffic and mature. Mol. Biol. Cell.

[212] Rowland A.A., Chitwood P.J., Phillips M.J., Voeltz G.K. (2014). ER contact sites define the position and timing of endosome fission. Cell.

[213] Dong R., Saheki Y., Swarup S., Lucast L., Harper J.W., De Camilli P. (2016). Endosome-ER contacts control actin nucleation and retromer function through VAP-dependent regulation of PI4P. Cell.

[214] Eden E.R., White I.J., Tsapara A., Futter C.E. (2010). Membrane contacts between endosomes and ER provide sites for PTP1B-epidermal growth factor receptor interaction. Nat. Cell Biol..

[215] Pfisterer S.G., Peranen J., Ikonen E. (2016). LDL-cholesterol transport to the endoplasmic reticulum: Current concepts. Curr. Opin. Lipidol..

[216] Haj F.G., Verveer P.J., Squire A., Neel B.G., Bastiaens P.I. (2002). Imaging sites of receptor dephosphorylation by PTP1B on the surface of the endoplasmic reticulum. Science.

[217] Ponting C.P., Aravind L. (1999). START: A lipid-binding domain in StAR, HD-ZIP and signalling proteins. Trends Biochem. Sci..

[218] Tsujishita Y., Hurley J.H. (2000). Structure and lipid transport mechanism of a StAR-related domain. Nat. Struct. Biol..

[219] Gulyás G., Sohn M., Kim Y.J., Várnai P., Balla T. (2020). ORP3 phosphorylation regulates phosphatidylinositol 4-phosphate and Ca(2+) dynamics at plasma membrane-ER contact sites. J. Cell Sci..

[220] Johansson M., Lehto M., Tanhuanpaa K., Cover T.L., Olkkonen V.M. (2005). The oxysterol-binding protein homologue ORP1L interacts with Rab7 and alters functional properties of late endocytic compartments. Mol. Biol. Cell.

[221] Du X., Kumar J., Ferguson C., Schulz T.A., Ong Y.S., Hong W., Prinz W.A., Parton R.G., Brown A.J., Yang H. (2011). A role for oxysterol-binding protein-related protein 5 in endosomal cholesterol trafficking. J. Cell Biol..

[222] Ochieng J., Pratap S., Khatua A.K., Sakwe A.M. (2009). Anchorage-independent growth of breast carcinoma cells is mediated by serum exosomes. Exp. Cell Res..

[223] Duijvesz D., Burnum-Johnson K.E., Gritsenko M.A., Hoogland A.M., Vredenbregt-van den Berg M.S., Willemsen R., Luider T., Pasa-Tolic L., Jenster G. (2013). Proteomic profiling of exosomes leads to the identification of novel biomarkers for prostate cancer. PLoS ONE.

[224] Forterre A., Jalabert A., Berger E., Baudet M., Chikh K., Errazuriz E., De Larichaudy J., Chanon S., Weiss-Gayet M., Hesse A.M. (2014). Proteomic analysis of C2C12 myoblast and myotube exosome-like vesicles: A new paradigm for myoblast-myotube cross talk?. PLoS ONE.

[225] Sharma S., Alharbi M., Kobayashi M., Lai A., Guanzon D., Zuniga F., Ormazabal V., Palma C., Scholz-Romero K., Rice G.E. (2018). Proteomic analysis of exosomes reveals an association between cell invasiveness and exosomal bioactivity on endothelial and mesenchymal cell migration in vitro. Clin. Sci. (Lond.).

[226] Harel A., Forbes D.J. (2004). Importin beta: Conducting a much larger cellular symphony. Mol. Cell.

[227] Soderholm J.F., Bird S.L., Kalab P., Sampathkumar Y., Hasegawa K., Uehara-Bingen M., Weis K., Heald R. (2011). Importazole, a small molecule inhibitor of the transport receptor importin-beta. ACS Chem. Biol..

[228] Zhou T., Li S., Zhong W., Vihervaara T., Beaslas O., Perttila J., Luo W., Jiang Y., Lehto M., Olkkonen V.M. (2011). OSBP-related protein 8 (ORP8) regulates plasma and liver tissue lipid levels and interacts with the nucleoporin Nup62. PLoS ONE.

[229] Panté N., Kann M. (2002). Nuclear pore complex is able to transport macromolecules with diameters of about 39 nm. Mol. Biol. Cell.

[230] Zhang H.G., Cao P., Teng Y., Hu X., Wang Q., Yeri A.S., Zhuang X., Samykutty A., Mu J., Deng Z.B. (2016). Isolation, identification, and characterization of novel nanovesicles. Oncotarget.

[231] Burns L.T., Wente S.R. (2012). Trafficking to uncharted territory of the nuclear envelope. Current Opin. Cell Biol..

[232] Chaumet A., Wright G.D., Seet S.H., Tham K.M., Gounko N.V., Bard F. (2015). Nuclear envelope-associated endosomes deliver surface proteins to the nucleus. Nature Commun..

[233] Rappa G., Green T.M., Lorico A. (2014). The nuclear pool of tetraspanin CD9 contributes to mitotic processes in human breast carcinoma. Mol. Cancer Res..

[234] Nunukova A., Neradil J., Skoda J., Jaros J., Hampl A., Sterba J., Veselska R. (2015). Atypical nuclear localization of CD133 plasma membrane glycoprotein in rhabdomyosarcoma cell lines. Int. J. Mol. Med..

[235] Cantile M., Collina F., D’Aiuto M., Rinaldo M., Pirozzi G., Borsellino C., Franco R., Botti G., Di Bonito M. (2013). Nuclear localization of cancer stem cell marker CD133 in triple-negative breast cancer: A case report. Tumori.

[236] Giri D.K., Ali-Seyed M., Li L.Y., Lee D.F., Ling P., Bartholomeusz G., Wang S.C., Hung M.C. (2005). Endosomal transport of ErbB-2: Mechanism for nuclear entry of the cell surface receptor. Mol. Cell Biol..

[237] De Angelis Campos A.C., Rodrigues M.A., de Andrade C., de Goes A.M., Nathanson M.H., Gomes D.A. (2011). Epidermal growth factor receptors destined for the nucleus are internalized via a clathrin-dependent pathway. Biochem. Biophys. Res. Commun..

[238] Packham S., Warsito D., Lin Y., Sadi S., Karlsson R., Sehat B., Larsson O. (2015). Nuclear translocation of IGF-1R via p150(Glued) and an importin-beta/RanBP2-dependent pathway in cancer cells. Oncogene.

[239] Papadopoulos N., Lennartsson J., Heldin C.H. (2018). PDGFRbeta translocates to the nucleus and regulates chromatin remodeling via TATA element-modifying factor 1. J. Cell Biol.

[240] Wang Y.N., Wang H., Yamaguchi H., Lee H.J., Lee H.H., Hung M.C. (2010). COPI-mediated retrograde trafficking from the Golgi to the ER regulates EGFR nuclear transport. Biochem. Biophys. Res. Commun..

[241] Brand T.M., Iida M., Li C., Wheeler D.L. (2011). The nuclear epidermal growth factor receptor signaling network and its role in cancer. Discov. Med..

[242] Shah P., Chaumet A., Royle S.J., Bard F.A. (2019). The NAE pathway: Autobahn to the nucleus for cell surface receptors. Cells.

[243] Wang Y.-N., Yamaguchi H., Hsu J.-M., Hung M.-C. (2010). Nuclear trafficking of the epidermal growth factor receptor family membrane proteins. Oncogene.

[244] Lo H.-W., Ali-Seyed M., Wu Y., Bartholomeusz G., Hsu S.-C., Hung M.-C. (2006). Nuclear-cytoplasmic transport of EGFR involves receptor endocytosis, importin β1 and CRM1. J. Cell Biochem..

[245] Stachowiak M.K., Maher P.A., Stachowiak E.K. (2007). Integrative nuclear signaling in cell development--a role for FGF receptor-1. DNA Cell Biol..

[246] Liao H.-J., Carpenter G. (2007). Role of the Sec61 translocon in EGF receptor trafficking to the nucleus and gene expression. Mol. Biol. Cell.

[247] Wurdinger T., Gatson N.N., Balaj L., Kaur B., Breakefield X.O., Pegtel D.M. (2012). Extracellular vesicles and their convergence with viral pathways. Adv. Virol..

[248] Meckes D.G.J. (2015). Exosomal communication goes viral. J. Virol..

[249] Nolte E., Cremer T., Gallo R.C., Margolis L.B. (2016). Extracellular vesicles and viruses: Are they close relatives?. Proc. Natl. Acad. Sci. USA.

[250] Narayanan A., Iordanskiy S., Das R., Van Duyne R., Santos S., Jaworski E., Guendel I., Sampey G., Dalby E., Iglesias-Ussel M. (2013). Exosomes derived from HIV-1-infected cells contain trans-activation response element RNA. J. Biol. Chem..

[251] Meckes D.G.J., Gunawardena H.P., Dekroon R.M., Heaton P.R., Edwards R.H., Ozgur S., Griffith J.D., Damania B., Raab-Traub N. (2013). Modulation of B-cell exosome proteins by gamma herpesvirus infection. Proc. Natl. Acad. Sci. USA.

[252] Gould S.J., Booth A.M., Hildreth J.E. (2003). The Trojan exosome hypothesis. Proc. Natl. Acad. Sci. USA.

[253] Feng Z., Hensley L., McKnight K.L., Hu F., Madden V., Ping L., Jeong S.H., Walker C., Lanford R.E., Lemon S.M. (2013). A pathogenic picornavirus acquires an envelope by hijacking cellular membranes. Nature.

[254] Bukong T.N., Momen-Heravi F., Kodys K., Bala S., Szabo G. (2014). Exosomes from hepatitis C infected patients transmit HCV infection and contain replication competent viral RNA in complex with Ago2-miR122-HSP90. PLoS Pathog..

[255] Van Dongen H.M., Masoumi N., Witwer K.W., Pegtel D.M. (2016). Extracellular vesicles exploit viral entry routes for cargo delivery. Microbiol. Mol. Biol. Rev..

[256] Harrison S.C. (2015). Viral membrane fusion. Virology.

[257] Hofemeister H., O’Hare P. (2008). Nuclear pore composition and gating in herpes simplex virus-infected cells. J. Virol..

[258] Malik P., Tabarraei A., Kehlenbach R.H., Korfali N., Iwasawa R., Graham S.V., Schirmer E.C. (2012). Herpes simplex virus ICP27 protein directly interacts with the nuclear pore complex through Nup62, inhibiting host nucleocytoplasmic transport pathways. J. Biol. Chem..

